# Non-Exponential ^1^H and ^2^H NMR Relaxation and Self-Diffusion in Asphaltene-Maltene Solutions

**DOI:** 10.3390/molecules26175218

**Published:** 2021-08-28

**Authors:** Kevin Lindt, Bulat Gizatullin, Carlos Mattea, Siegfried Stapf

**Affiliations:** Department of Technical Physics II/Polymer Physics, Institute of Physics, Faculty of Mathematics and Natural Science, Ilmenau University of Technology, P.O. Box 100565, D-98684 Ilmenau, Germany; bulat.gizatullin@tu-ilmenau.de (B.G.); carlos.mattea@tu-ilmenau.de (C.M.)

**Keywords:** asphaltenes, maltenes, NMR, relaxation, diffusion

## Abstract

The distribution of NMR relaxation times and diffusion coefficients in crude oils results from the vast number of different chemical species. In addition, the presence of asphaltenes provides different relaxation environments for the maltenes, generated by steric hindrance in the asphaltene aggregates and possibly by the spatial distribution of radicals. Since the dynamics of the maltenes is further modified by the interactions between maltenes and asphaltenes, these interactions—either through steric hindrances or promoted by aromatic-aromatic interactions—are of particular interest. Here, we aim at investigating the interaction between individual protonic and deuterated maltene species of different molecular size and aromaticity and the asphaltene macroaggregates by comparing the maltenes’ NMR relaxation ($T_{1}$ and $T_{2}$) and translational diffusion (*D*) properties in the absence and presence of the asphaltene in model solutions. The ratio of the average transverse and longitudinal relaxation rates, describing the non-exponential relaxation of the maltenes in the presence of the asphaltene, and its variation with respect to the asphaltene-free solutions are discussed. The relaxation experiments reveal an apparent slowing down of the maltenes’ dynamics in the presence of asphaltenes, which differs between the individual maltenes. While for single-chained alkylbenzenes, a plateau of the relaxation rate ratio was found for long aliphatic chains, no impact of the maltenes’ aromaticity on the maltene–asphaltene interaction was unambiguously found. In contrast, the reduced diffusion coefficients of the maltenes in presence of the asphaltenes differ little and are attributed to the overall increased viscosity.

## 1. Introduction

Crude oils are complex fluids consisting of thousands of chemical species of different structures and sizes, which can be fractionated into saturates, aromatics, resins, and asphaltenes (SARA) based on their solubility in different solvents. Saturates, aromatics and resins are defined as maltenes and are soluble in n-alkane solvents, for example, n-pentane or n-heptane, whereas the molecule fraction insoluble in n-alkane solvents is defined as asphaltenes. The presence of asphaltenes in crude oil can be the origin of severe issues related to production, refinery, and transportation, affecting the economic potential of the crude oil. The precipitation of asphaltenes, caused by changes in temperature, pressure, or the composition of the crude oil, can lead to fouled and clogged pipes in the extraction process or a blocked catalytic network in the refinery process, putting the process to a halt and requiring extensive cleaning. The asphaltene molecules themselves consist of polycyclic aromatic hydrocarbons (PAH) with alkyl side chains and contain heteroatoms like O, N, and S. Their tendency to self-aggregate distinguishes them from the other oil components and results, after the formation of nanoaggregates and clusters, in asphaltene macroaggregates, which are colloidally dispersed in the crude oil or a solvent until they precipitate as black, friable solids up to dense solid deposits [1].

To assess the risk of asphaltene precipitation and to choose the extraction parameters, one needs to gain insight into the conditions in the oil reservoir such as oil saturation or capillary pressures as well as the properties of the oil, i.e., its qualitative and quantitative composition, which ideally are obtained in situ downhole. Nowadays, nuclear magnetic resonance (NMR) techniques are well established among the standard downhole measurement techniques. This technique determines longitudinal ($T_{1}$) and transverse ($T_{2}$) relaxation times of the maltene nuclei, as well as the self-diffusion coefficients (*D*) of these species. The parameters are further characterized by a broad distribution due to the complexity of the crude oil. The relaxation times are dominated by molecular rotations, eventually modulated by diffusion, whereas the diffusion coefficient measured by NMR reflects the translational motion on typical scales of µm. Both quantities are related to each other and depend on different ways on the viscosity, which is a result of the oil composition. This was investigated in detail for linear alkanes [2,3]. For the increased complexity in crude oils due to the vast amount of components, two-dimensional techniques that correlate two of the three parameters were applied to assess the oil composition [4]. Most references agree that one average relaxation time be considered for a particular maltene, and empiric correlations attempt to define the maltene distribution in crude oil, e.g., by the average $T_{1}/T_{2}$ ratio in a $T_{1}$-$T_{2}$ map.

The situation is further complicated by the presence of asphaltenes, which provide an additional relaxation mechanism due to geometrical hindrance or unpaired electrons from ions such as VO^2+^, which are present in the form of vanadyl porphyrins in asphaltene aggregates [5,6,7], or persistent free radicals [8,9,10] and therefore affect the relaxation of the maltenes significantly, while the diffusion properties are hardly affected [4]. As mentioned above, the possibility of non- or multiexponential relaxation for maltenes is frequently not considered in asphaltene-containing crude oils, and similar correlations are being established while neglecting the influence of the presence of asphaltenes on the maltenes’ behavior.

Our previous studies [11,12] showed a significant (factor 20) difference in the $T_{1}/T_{2}$ ratios of fluorinated aromatic ring molecules (higher ratio) (benzene-f${}_{6}$ and toluene-f${}_{8}$) and aliphatic chain molecules (lower ratio) (octane-f${}_{18}$ and pentadecane-f${}_{32}$) in an oil containing 13 wt% asphaltenes, which is much smaller in an asphaltene-free resinic oil and vanishes in an asphaltene- and resin-free oil. This difference in the $T_{1}/T_{2}$ ratio persists in a solution of chloroform-d and 3 vol% of the tracer molecule (benzene-f${}_{6}$ and octane-f${}_{18}$) containing 10 wt% asphaltenes [13], extracted from the previous investigated asphaltenic oil. Furthermore, a strong dispersion of $T_{1}$ at Larmor frequencies between $10^{6}$ Hz and $10^{8}$ Hz was observed for the aromatic molecules in the asphaltenic oil, which is in accordance with the $T_{1}$ dispersion of the maltene mixtures present in crude oils containing asphaltenes [14,15,16]. In contrast, a significantly weaker dispersion is observed for octane-f${}_{18}$ in the asphaltenic oil as well as for the aromatic molecules in the asphaltene-free oil [11]. Additionally, it was found [17] that in comparison with saturated ${}^{1}$H fraction, an aromatic ${}^{1}$H fraction of the crude oil exhibits stronger interaction with asphaltene molecules. Furthermore, molecular dynamics and NMR relaxation of maltenes in the proximity of asphaltenes cannot be characterized by a simple model as in the case of only high molecular weight compounds solution even containing paramagnetic impurities [18].

The aim of this work is to study the maltene–asphaltene interaction for individual maltenes rather than a mixture of maltenes. NMR relaxometry and diffusometry methods are used to access the microscopic and macroscopic maltene motion. Since the non-monoexponential relaxation of maltenes in crude oils persists in the absence of asphaltenes, its origin is located in the large number of different maltenes present in crude oils [19]. On the other hand, the broadening of the $T_{1}$ distribution for lower frequencies in asphaltenic crude oils indicates maltene populations in different relaxation environments as an additional cause for their non-monoexponential relaxation in the presence of asphaltenes. The investigation of individual maltenes eliminates the non-monoexponential decay due to different maltene species and thus allows us to study the influence of the asphaltenes on the maltene’s relaxation. The focus on individual maltenes rather than a maltene mixture further opens the possibility to study the influence of the maltene’s size, shape, and aromaticity on the maltene–asphaltene interaction.

## 2. Theory

For protons, the most dominant NMR relaxation mechanism is the dipolar coupling between spin-containing nuclei, which consists of an intra- and an intermolecular contribution. However, in the presence of unpaired electrons, e.g., due to free radicals and paramagnetic ions in asphaltenes, the relaxation may be dominated by the dipolar coupling between the nucleus and the free electron. The relaxation, i.e., the return of the total magnetization towards its equilibrium, is enabled by the modulation of the local magnetic field environment of the nucleus. This field modulation occurs due to the molecular rotational and translational motion and is therefore influenced by the viscosity of the solution and the molecule’s size.

The intermolecular part of the dipole–dipole interaction is predominantly influenced by the translational motion of the molecule since the interacting nuclei are located at different molecules, while the intramolecular dipolar coupling occurs between nuclei on the same molecule. For small molecules, the intramolecular part is therefore dominated by the rotational motion of the molecule, while for larger molecules the internal motions can gain in importance. The motion relevant for the dipolar coupling between a nucleus and a free electron depends on whether the unpaired electron is located on a different molecule or within the molecule hosting the interacting nucleus. Due to the presence of an electric quadrupole moment, the dominant relaxation mechanism for deuterons is the quadrupolar coupling, which is the interaction of the quadrupolar moment with the electric field gradient at the site of the nucleus. The quadrupolar coupling is a very strong relaxation mechanism and the dipolar coupling becomes negligible for relaxation so that the relaxation of deuterons solely reflects the rotational motion of the molecule. The relaxation rates $R_{1,2}$ of the molecule result additively from the different contributions of the relaxation mechanisms:

(1) $$\begin{array}{cc} R_{1,2}^{{}^{1}H}=\underset{\begin{array}{c} \mathrm{intra}-\;\mathrm{and}\;\mathrm{intermolecular}\\ \mathrm{contribution}\;\mathrm{to}\;\mathrm{the}\;\mathrm{dipolar}\\ \mathrm{coupling}\;\mathrm{between}\;\mathrm{nuclei} \end{array}}{\underbrace{R_{1,2}^{D,intra}+R_{1,2}^{D,inter}}}+ & \underset{\begin{array}{c} \mathrm{dipolar}\;\mathrm{coupling}\\ \mathrm{between}\;\mathrm{nuclei}\;\mathrm{and}\\ \mathrm{unpaired}\;\mathrm{electrons} \end{array}}{\underbrace{R_{1,2}^{D,elec}}} \end{array}$$

(2) $$\begin{array}{cc} R_{1,2}^{{}^{2}H}=\;\underset{\begin{array}{c} \mathrm{quadrupolar}\;\mathrm{coupling}\\ \mathrm{between}\;\mathrm{nuclei} \end{array}}{\underbrace{R_{1,2}^{Q}}}+ & \underset{\begin{array}{c} \mathrm{dipolar}\;\mathrm{coupling}\\ \mathrm{between}\;\mathrm{nuclei}\;\mathrm{and}\\ \mathrm{unpaired}\;\mathrm{electrons} \end{array}}{\underbrace{R_{1,2}^{D,elec}}} \end{array}$$

In case of the protonic maltenes, the reference solution consists of only 5 vol% of proton bearing molecules, so that the intermolecular contribution to the dipolar coupling can be neglected. This no longer applies for the asphaltene-containing solution since asphaltenes are mainly composed of carbons and protons (n(${}^{1}$H)/n(C) ≈ 1...1.3 [20,21]) and the asphaltene concentration (≈ 17 wt%) is quite high (asphaltene concentration in crude oils ranges from 0 wt% up to approximately 20 wt% [21,22,23]).

As mentioned above, the relaxation requires a fluctuation of the local magnetic field in the environment of the nucleus, e.g., caused by the molecular motion. The fluctuation of the local magnetic field is described by an autocorrelation function, and its normalized Fourier transform, the reduced spectral density I(ω), contains the Larmor frequency dependence of the relaxation rates *R* [24,25]:

(3) $$\begin{array}{c} \begin{array}{cc} R_{1}^{D,II}= & \frac{1}{5}C_{D}\left[I\left(\omega_{I}\right)+4I\left(2\omega_{I}\right)\right]\\ R_{2}^{D,II}= & \frac{1}{10}C_{D}\left[3I\left(0\right)+5I\left(\omega_{I}\right)+2I\left(2\omega_{I}\right)\right] \end{array} \end{array}$$

(4) $$\begin{array}{c} \begin{array}{cc} R_{1}^{D,IS}= & \frac{1}{15}C_{D}\left[I\left(\omega_{I}-\omega_{S}\right)+3I\left(\omega_{I}\right)+6I\left(\omega_{I}+\omega_{S}\right)\right]\\ R_{2}^{D,IS}= & \frac{1}{30}C_{D}\left[4I\left(0\right)+I\left(\omega_{I}-\omega_{S}\right)+3I\left(\omega_{I}\right)+6I\left(\omega_{S}\right)+6I\left(\omega_{I}+\omega_{S}\right)\right] \end{array} \end{array}$$

$$\begin{array}{c} \mathrm{with}\;\;\;C_{D}={\left(\frac{\mu_{0}}{4\pi }\right)}^{2}\gamma_{I}^{2}\gamma_{S}^{2}\hbar^{2}S\left(S+1\right)C_{int},\;\;\;C_{int}=\left\{\begin{array}{cc} \frac{1}{r^{6}} & \mathrm{for}\;\mathrm{intramolecular}\;\mathrm{coupling}\\ 72\frac{N_{I}}{d^{3}} & \mathrm{for}\;\mathrm{intermolecular}\;\mathrm{coupling} \end{array}\right. \end{array}$$

and the Larmor frequency $\omega =\gamma B_{0}$. The prefactor $C_{D}$ incorporates the vacuum permeability $\mu_{0}$, the gyromagnetic ratio γ of the considered nucleus ($\gamma_{I}$) and the nucleus coupled to it ($\gamma_{S}$), as well as the spin number of the coupled nucleus *S*. In the homonuclear case (Equation (3)), where two nuclei of the same type are coupled together, S=I applies. The relaxation rate for the nucleus–electron interaction can be obtained from the equations for heteronuclear coupling (Equation (4)). The difference between intra- and intermolecular contribution lies in the prefactor $C_{int}$, as well as in the shape of the reduced spectral density (see Equations (6) and (7)). The prefactor $C_{int}$ contains the intramolecular nuclei–nuclei distance *r* or, in case of intermolecular coupling, the distance of closest approach *d* between the interacting nuclei and *N*, the number of spins *I* per volume.

The relaxation rates for the quadrupolar relaxation [25] in case of, e.g., deuterons differs only by the prefactors from the ones describing the intramolecular, homonuclear dipolar coupling (Equations (3) and (6)):

(5) $$\begin{array}{c} \begin{array}{cc} R_{1}^{Q}= & \frac{3}{80}C_{Q}\left[I\left(\omega_{I}\right)+4I\left(2\omega_{I}\right)\right]\\ R_{2}^{Q}= & \frac{3}{160}C_{Q}\left[3I\left(0\right)+5I\left(\omega_{I}\right)+2I\left(2\omega_{I}\right)\right] \end{array} \end{array}$$

$$\begin{array}{cc} \mathrm{with}\;\;\;C_{Q} & ={\left(\frac{e^{2}qQ}{\hbar }\right)}^{2}\left(1+\frac{\eta^{2}}{3}\right) \end{array}$$

The first term of the quadrupolar prefactor $C_{Q}$ is known as quadrupole coupling constant, consisting of the electric field gradient *q* and the quadrupole moment *Q*, while the second term reflects the asymmetry of the electric field by the asymmetry parameter η.

The reduced spectral density contains, via the frequency dependence of the relaxation rates, the dependence on the molecular motion, which can be represented by a correlation time τ. The properties of molecular motion are not only reflected in different correlation times, but also in the functional form of the reduced spectral density. For isotropic rotation, this function is described by a Lorentzian [25]

(6) $$\begin{array}{c} I_{intra}\left(\omega \right)=I_{rot}\left(\omega \right)=\frac{2\tau_{rot}}{1+\omega^{2}\tau_{rot}^{2}}. \end{array}$$

The Lorentzian shape of the reduced spectral density function results from an exponential correlation function, which derives from the assumption of random motion. Assuming the force-free-hard-sphere model, the reduced spectral density of translational motion, which dominates the intermolecular relaxation, can be expressed as [26]

(7) $$\begin{array}{c} I_{inter}\left(\omega \right)=I_{trans}\left(\omega \right)=\int_{0}^{\infty }\frac{u^{2}}{81+9u^{2}-2u^{4}+u^{6}}\frac{u^{2}\tau_{trans}}{u^{4}+\omega^{2}\tau_{trans}^{2}}du \end{array}$$

$$\begin{array}{c} \mathrm{with}\;\;\;\tau_{trans}=\frac{d^{2}}{D_{IS}}, \end{array}$$

where *d* denotes the distance of closest approach of the interacting species, *D* the relative translational diffusion coefficient as a sum of the diffusion coefficients of the spin-bearing molecules $D_{IS}=D_{I}+D_{S}$, and *u* a dimensionless integration variable.

In the BPP model, i.e., when dipolar relaxation is dominated by isotropic rotation, the frequency dependence of $T_{1}$ is proportional to $\omega^{2}$ until $T_{1}$ becomes independent of the Larmor frequency in the extreme narrowing limit, when ωτ≪0.707, and $T_{1}=T_{2}$, e.g., in low viscosity liquids. In the region ωτ≫0.707, which can be found, for example, for high viscosity liquids, the ratio of $T_{1}$ and $T_{2}$ grows with increasing correlation times. The extreme narrowing region reflects therefore motions with correlation times faster than 1/ω, i.e., fast dynamics, while the region of large $T_{1}/T_{2}$ ratios reflects motions with correlation times comparable to or slower than 1/ω, i.e., slow dynamics. This is true for protons and deuterons in rotation dominated molecules since the shape of Equation (3) and Equation (5) do not differ.

The free electrons present in asphaltenes provide a highly effective ($\omega_{e}\approx 658\;\omega_{{}^{1}H}$) variant of the dipolar relaxation to the maltenes, as the maltenes’ nuclear dipole interacts with a dipole of the free electron. Since the interacting dipoles are located on different molecules, the intermolecular interaction between maltenes and asphaltenes becomes important for the maltenes’ relaxation. In addition, the self-aggregated structures of asphaltene molecules in crude oil or asphaltene-solvent solutions interfere with the motions of the maltenes, slowing them down so that the reorientations of the maltenes no longer fulfill the extreme narrowing limit, and $T_{1}/T_{2}$ ratios larger than one and a frequency dependence of $T_{1}$ are observed [27]. Investigations of maltenes’ relaxation in the presence of asphaltenes show faster relaxation with higher asphaltene content and a stronger increase in the transverse than the longitudinal relaxation rate [13,19]. The latter observation indicates significant importance of the decreased maltene mobility due to contact and entanglement with the asphaltene structures, as the proximity to free electrons influences the longitudinal and transverse relaxation equally. The BPP assumption of random motion is therefore not valid for these systems, and models taking a correlated motion into account were developed to explain the relaxation behavior of the maltenes.

The current understanding of maltene–asphaltene interaction is based on the work of Zielinski et al. and Korb et al., who both found a strong $T_{1}$ dispersion for the maltenes in asphaltenic-resinic, but not in asphaltene-free crude oils [14,15]. The reorientations of the maltene molecules in these asphaltene-free oils are hence fast enough to fulfill the extreme narrowing limit, i.e., $T_{1}$ is frequency independent, while the presence of asphaltenes results in slower maltene reorientations, which do not fulfill the extreme narrowing limit anymore. Since the viscosities of the studied asphaltene-free and asphaltenic oils did not differ substantially, the relaxation dispersion is an effect of the maltene–asphaltene interaction, which couples the normally fast motion of the maltenes to the slow motion of the asphaltene macrostructures. Although the asphaltene content of the oil is the dominating factor for the relaxation dispersion, the influence of the oil’s total composition is not negligible. In case of a low resin content (< 15 wt) and a lack of asphaltenes, often no dispersion is found (for example sample 2 in [15], oil A-D in [14], but not oil 10 in [28]), while in crude oils with a similar asphaltene content, a stronger dispersion is found for the more resinic oil (compare oils E and J, as well as G and I in [14]). The influence of the resin on the dispersion may be seen in [14] (although the composition of the used oil, besides its asphaltene content, is unknown), where the removal of the asphaltene content of an oil results in a partial disappearance of the dispersion below 1 MHz, which is consistent with asphaltenes forming the largest structures and hence are the slowest components in crude oils. Furthermore, in the asphaltenic-resinic oils, the shape of the $T_{1}$ distribution becomes narrower with increasing frequency, while this is not observed in the asphaltene-free oils. Korb et al. were able to fit the $T_{1}$ distribution with a bimodal log-normal distribution at low frequencies and a single log-normal distribution at high frequencies (15 MHz) according to a model based on the translational motion of maltenes on a locally flat surface containing unpaired electrons. While the principal distribution of $T_{1}$ (and also $T_{2}$) is attributed to the vast amount of different maltenes present in the crude oil, Korb and coworkers concluded from their bimodal log-normal distribution fit different relaxation environments of the maltenes, which depend on their proximity to the asphaltene macrostructures. Although the models of Zielinski and Korb differ slightly regarding the detailed interaction between the maltenes and the asphaltenes, the overall concept is similar: The relaxation behavior of the maltenes is caused by their motion through the slowly rotating porous asphaltene macrostructures. In contact with the asphaltenes, the maltenes diffusion is correlated, while the diffusion between the asphaltene structures is only influenced by the global composition of the solution, i.e., its viscosity. Furthermore, fast exchange between the surface region and the bulk region is assumed.

Since the model is developed from the investigations of crude oils, i.e., a mixture of various different maltenes, descriptions of the interaction of individual maltene species with the asphaltene structures are limited in the literature. However, it is reasonable to assume that the size of the maltene is an important parameter affecting the contact time and the maltene-asphaltene distance, i.e., the distance between the maltenes’ nuclei and corresponding spins on the asphaltenes, as it influences possible entanglements and the trapping of maltene molecules in the asphaltene structures. For example, the increase in relaxation rates was found to be larger for longer and less mobile hydrocarbon chains with increasing asphaltene concentration [19], though the contact time between the maltenes and asphaltenes is still short, as the overall diffusion of the maltenes is not severely hindered [4,16,19].

In addition to a purely steric interaction between the maltenes and the asphaltenes, interactions between aromatic parts of the maltenes and the asphaltenes are conceivable and may contribute to the contact time and the minimum maltene–asphaltene distance. These aromatic interactions, often referred to as π-stacking, describe attractive forces between aromatic ring molecules, due to interactions between their aromatic π electron clouds. The result can be a rather stable structure, as it is for example reported for the benzene dimer (dissociation energy (moderate hydrogen bonds: $D_{0}=\left(4-15\right)\mathrm{kcal}/\mathrm{mol}$ [29], van der Waals bonds: $D_{0}≲1\mathrm{kcal}/\mathrm{mol}$) $D_{0}=\left(2.0-2.7\right)\mathrm{kcal}/\mathrm{mol}$) [30,31], though the importance of π-stacking, that means the face-centered stacking arrangement of aromatic molecules, for aromatic–aromatic interactions is still under discussion [32]. Nevertheless, π-π interactions are considered as an important interaction among others responsible for asphaltene aggregation and precipitation [33,34,35,36]. Recently, an unusual type of parallel π-stacking, the so-called pancake bonding, which occurs between radicals with highly delocalized π electrons, as they occur in asphaltenes, gained attention with regard to the aggregation of asphaltenes [37]. The pancake bonding differs from the π-stacking by a stronger interaction, closer contact distances, and a preferred orientation for direct atom-to-atom overlapping due to an energy lowering overlapping of the singly occupied molecular orbitals of the radicals [38].

Some studies have addressed the interactions of maltenes and asphaltenes by density functional theory [39,40,41] and molecular dynamics simulations [42,43], and experimental studies have investigated the aggregation ability of asphaltenes in different solvents [44,45,46]. Although some of these studies found evidence of a stronger interaction between asphaltenes and aromatic molecules, the aromatic–aromatic interactions do not appear to be exclusively responsible for the asphaltene–maltene interactions, as strong anionic, polar and acid-base interactions were also found.

While the direct comparison of the shape of the relaxation decays is a straightforward method to identify monoexponential and non-monoexponential relaxations, the $T_{1}/T_{2}$ ratio allows an estimation of the strength of restriction the asphaltene structures impose on the maltene’s motion. Since the $T_{1}/T_{2}$ ratio is close to unity in the extreme narrowing limit, but larger for motions slower than the inverse of the Larmor frequency, a higher ratio indicates a slower motion. Furthermore, the $T_{1}/T_{2}$ ratio is an often used parameter in borehole analysis to classify the extracted oil. It is obtained from a line in the $T_{1}$-$T_{2}$ plot parallel to $T_{1}=T_{2}$ and allows summarizing properties of a mixture of substances by a common parameter.

## 3. Materials and Methods

Maltene molecules and solvents were purchased from different vendors (see Appendix A) and were used without further purification or degassing. Benzene and naphthalene were chosen to represent typical aromatic molecules. To investigate the influence of the aromaticity of the maltenes on the interaction with the asphaltene, the set is extended by n-decane, a saturated hydrocarbon, and the cycloalkanes cyclohexane and decalin. In addition, several alkylbenzenes (toluene, ethylbenzene, propylbenzene, butylbenzene, and decylbenzene) were selected to explore a possible impact of a side chain of increasing length attached to an aromatic core on the maltene–asphaltene interactions. Furthermore, fully deuterated (benzene-d${}_{6}$, naphthalene-d${}_{8}$, decalin-d${}_{18}$, cyclohexane-d${}_{12}$, nonane-d${}_{20}$, toluene-d${}_{8}$, ethylbenzene-d${}_{10}$) variants were included to study directly the impact of the asphaltene on the rotational motion of the maltenes since the relaxation of the deuterated maltenes is expected to be dominated only by intramolecular contributions. In addition, due to the lack of deuterons in asphaltenes, there is no signal overlap of the maltenes and asphaltenes and therefore the sole maltene dynamic is observed.

### 3.1. Sample Preparation

The asphaltene-free reference solutions consist of 95 vol% of a solvent, benzene-d${}_{6}$ in case of the protonic maltenes and benzene for the deuterated variants, and 5 vol% of the maltene. As the impact of the presence of asphaltenes on the relaxation times of the maltene molecules increases with the asphaltene concentration [13,19], a high asphaltene concentration is desirable. Hence, an asphaltene concentration of 150 g per liter of reference solution (≈ 17 wt%) is used for the asphaltene solutions. This concentration is well above the concentration where asphaltene cluster formation starts [47,48,49].

The asphaltene was provided by Schlumberger Doll Research and was extracted from oil A13 used in references [11,12]. It is the same as in reference [13], where its radical content was determined by EPR to ($145\pm 7\times 10^{14}$ spins per mg.

The lack of a sufficient amount of the asphaltene and the large set of samples required a sequential sample preparation and the reuse of the asphaltene. Therefore, the asphaltene solutions with the protonic maltenes were prepared directly in the 5 mm NMR tube, which was flame sealed afterward. After the measurement, the samples were reopened and kept in an oven at 100 °C until the liquid components had evaporated (checked by weight) and only the solid asphaltene remained. The asphaltene is unchanged by this process and due to the in-tube preparation the amount of asphaltene in the tube is known so that a new sample can be prepared in the reopened tube. The repetition of this process is limited by the minimum required length of the tube to fit into the spectrometer. In this work, the initial amount of asphaltene in a tube was used for two samples. Attention was paid that the subsequential samples consisted of chemically similar maltenes and solvents, e.g., the sample consisting of benzene-d${}_{6}$, toluene and asphaltene is followed by the sample consisting of benzene, toluene-d${}_{8}$ and asphaltene.

The in-tube preparation of the asphaltene samples was carried out in the following way. After the asphaltene (30 mg) was placed in the tube and filled up with the reference solution (200 μL), the tube was flame sealed. To assist the permeation process of the reference solution in the air-filled pores between the asphaltene flakes, especially in the case of the reused asphaltene, the sample was manually centrifuged for one minute. Afterward, the sample was held for two minutes on a laboratory shaker with 1000 rpm before being placed in an ultrasonic bath at room temperature for 30 min. The samples were stored for at least 12 h at 5 °C in the refrigerator before measurement.

### 3.2. Measurement and Evaluation

The experiments were carried out at room temperature at a magnetic field strength of 7.05 T with a Bruker Avance III 300. The spectral resolution obtained allows a distinction between aromatic and aliphatic nuclei (see Appendix B) so that their relaxation is evaluated separately. The inversion recovery (IR) and the Carr–Purcell–Meiboom–Gill (CPMG) pulse sequence were used to obtain the longitudinal ($T_{1}$) and transverse ($T_{2}$) recovery functions. The relaxation curves were either fitted with a single exponential function or a sum of two exponential functions of the form:

(8) $$\begin{array}{cc} S_{j}\left(t\right)=S_{\infty }+ & \sum_{i=1}^{n}A_{i}e^{-\frac{t}{T_{k_{i}}}}\;\;\;\mathrm{with}\;\;\;n=1,2\\ j=z,\;k=1\; & \mathrm{for}\;\mathrm{longitudinal}\;\mathrm{relaxation}(\mathrm{IR})\\ j=xy,\;k=2,\;\;S_{\infty }=0\; & \mathrm{for}\;\mathrm{transverse}\;\mathrm{relaxation}\;\left(\mathrm{CPMG}\right) \end{array}$$

The fit covers a range between $t_{0}$ and the loss of the signal below noise level, but with a maximum $t_{end}^{CPMG}=10s$ in case of the transverse relaxation decays. This results from a compromise regarding the echo time in the CPMG sequence, which was chosen as $\tau_{e}=1\mathrm{ms}$ in all $T_{2}$ experiments to ensure the comparability of the experiments with the asphaltene-free and the asphaltenic samples. The selected echo time is short enough to enable a good resolution of the signal decay in the asphaltenic samples, as well as long enough to capture at least the signal decay below 1/e in the asphaltene-free samples. The $90^{\circ }$ and $180^{\circ }$ pulse durations were 5.75 μs and 11.5 μs for the proton and 56 μs and 112 μs for the deuteron measurements, respectively.

Additionally, the diffusion coefficients of the protonic maltenes in the absence and presence of the asphaltene were determined with the Pulse Gradient Stimulated Echo (PGSTE) sequence using half-sine shaped gradient pulses, with a duration of δ=2ms and a separation between the gradient pulses of Δ=20ms. The gradient strength *g* was varied up to 1 T/m to determine the self-diffusion coefficients of the protonic maltenes. The time between the first two radio frequency pulses is about 4.2 ms. In the absence of asphaltene, the data were fitted with a single exponential function, while in the presence of asphaltene, a deviation from a monoexponential decay was observed at larger gradients *g*, which is attributed to the significantly slower diffusion of the asphaltene. The signal decay is therefore fitted in the following way:

(9) $$\begin{array}{c} S_{D}\left(g^{2}\right)=\sum_{i=1}^{n}A_{i}e^{-D_{i}4\pi^{2}\gamma_{{}^{1}H}^{2}\delta^{2}\left(\Delta -\frac{\delta }{3}\right)g^{2}}\;\;\;\mathrm{with}\;\;\;n=\left\{\begin{array}{cc} 1 & \mathrm{asphaltene}-\mathrm{free}\\ 2 & \mathrm{with}\;\mathrm{asphaltene} \end{array}\right. \end{array}$$

Since the measurements aimed at the determination of the diffusion coefficient of the maltenes, the obtained data is not sufficient to reliably determine the diffusion coefficient of the asphaltene in the different maltene solutions. Including $D_{2}$ as a free parameter in the fit results in an order of E−10 m^2^/s for the reasonable fits, while some decays could not be fitted. Therefore, the parameter $D_{2}$ was fixed to the fastest diffusion coefficient obtained for the aliphatic asphaltene protons in a solution consisting of 150 g/L asphaltene in benzene-d${}_{6}$ (see Section 4.4). The obtained fits describe the data well and provide reliable diffusion coefficients for the maltenes.

## 4. Results

The results of the experiments are evaluated by a direct comparison of the signal decay, as well as the relaxation time constants $T_{1}$ and $T_{2}$ and their respective relative weights $p_{1}$ and $p_{2}$. Furthermore, the mean relaxation rate constants $R_{1}$ and $R_{2}$ are computed to obtain a single variable classifying the longitudinal and transverse relaxation of the different samples.

(10) $$\begin{array}{c} {\bar{R}}_{k}=\sum_{i=1}^{n}p_{i}\;\cdot \;\frac{1}{T_{k_{i}}}\;\;\;\mathrm{with}\;\;k=1,2, \end{array}$$

where $p_{i}$ denotes the relative weight of the $T_{1_{i},2_{i}}$ component and is calculated from the amplitudes $A_{i}$ of the different exponential functions in the fit:

(11) $$\begin{array}{c} p_{i}=\frac{A_{i}}{\sum_{i=1}^{n}A_{i}}\;\;\;\mathrm{with}\;\;\;n=1,2\;\;\;\mathrm{and}\;\;\;\sum_{i=1}^{n}p_{i}=1. \end{array}$$

In addition, the mean relaxation rate constants enable the construction of the $R_{2}/R_{1}$ ratio, equivalent to the widely used $T_{1}/T_{2}$ ratio, known from borehole analysis to evaluate the crude oil regardless of the detailed form of the relaxation time constant distributions. The mean relaxation rate constant is chosen, since the relaxation rate constant is, unlike the relaxation time constant, directly proportional to the spectral density function (see Equations (3)–(5)) and therefore the mean relaxation rate constant is proportional to the mean spectral density function.

The mean relaxation rate constants are also used to enable the qualitative comparison of the relaxation decays of the different samples regarding their monoexponentiality. The time axis of the relaxation decay of each sample is rescaled with the corresponding mean relaxation rate constant

(12) $$\begin{array}{c} t^{*}={\bar{R}}_{1,2}\;\cdot \;t, \end{array}$$

so that $t^{*}$ reflects multiples of ${\bar{R}}_{1,2}^{-1}$. Furthermore, the signal is normalized according to:

(13) $$\begin{array}{c} S_{z}^{*}=\frac{S_{z}-S_{\infty }}{\sum_{i=1}^{n}A_{i}} \end{array}$$

(14) $$\begin{array}{c} S_{xy}^{*}=\frac{S_{xy}}{\sum_{i=1}^{n}A_{i}} \end{array}$$

**Figure 1:**
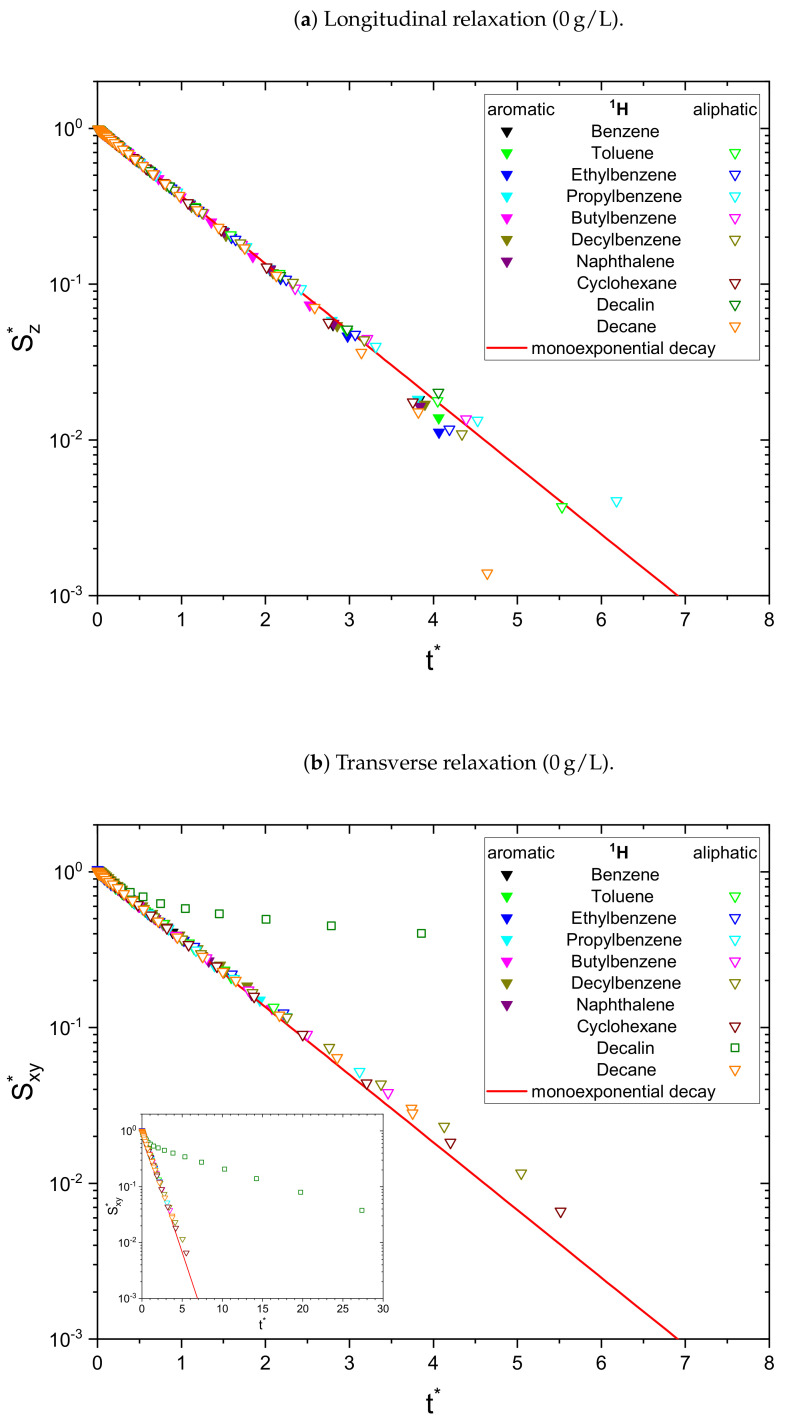
Longitudinal (**a**) and transverse (**b**) relaxation decays of the maltene protons in the asphaltene-free solution, consisting of 95 vol% benzene-d_6_ and 5 vol% of the investigated maltene. The inset in (**b**) shows the whole measured range of the relaxation decay of decalin. For a qualitative comparison, the time axis is rescaled (see Equation (12)) to reflect multiples of ${\bar{R}}_{1,2}^{-1}$ and the signal is normalized to unity at t* = 0 (see Equations (13) and (14)). The needed parameters were obtained from a monoexponential (∇) or biexponential (☐) fit.

As a result, the signal $S_{j}^{*}$ is normalized to unity at $t^{*}=0$ and all monoexponential decays collapse to $S_{j}^{*}=e^{-t^{*}}$ (see Figure 1a). Non-monoexponential decays differ from this relation, and the visualization of $\log S^{*}$ over $t^{*}$ allows the qualitative comparison of the monoexponentiality of the relaxation.

As mentioned in Section 3.2 the relaxation decays were fitted with a single exponential or a sum of two exponential functions to obtain the parameters $T_{k_{i}}$ and $A_{i}$. The decision between a monoexponential and biexponential fit is based on the obtained data, regarding which fit describes the observed relaxation decay sufficiently. In all cases of non-monoexponential decays, we identified a biexponential fit as sufficient and therefore did not attempt to fit a sum of more than two exponential functions. This does not mean that the relaxation consists of two components, but that it could be described sufficiently with the specified time constants.

### 4.1. Relaxation Decays

**Figure 2:**
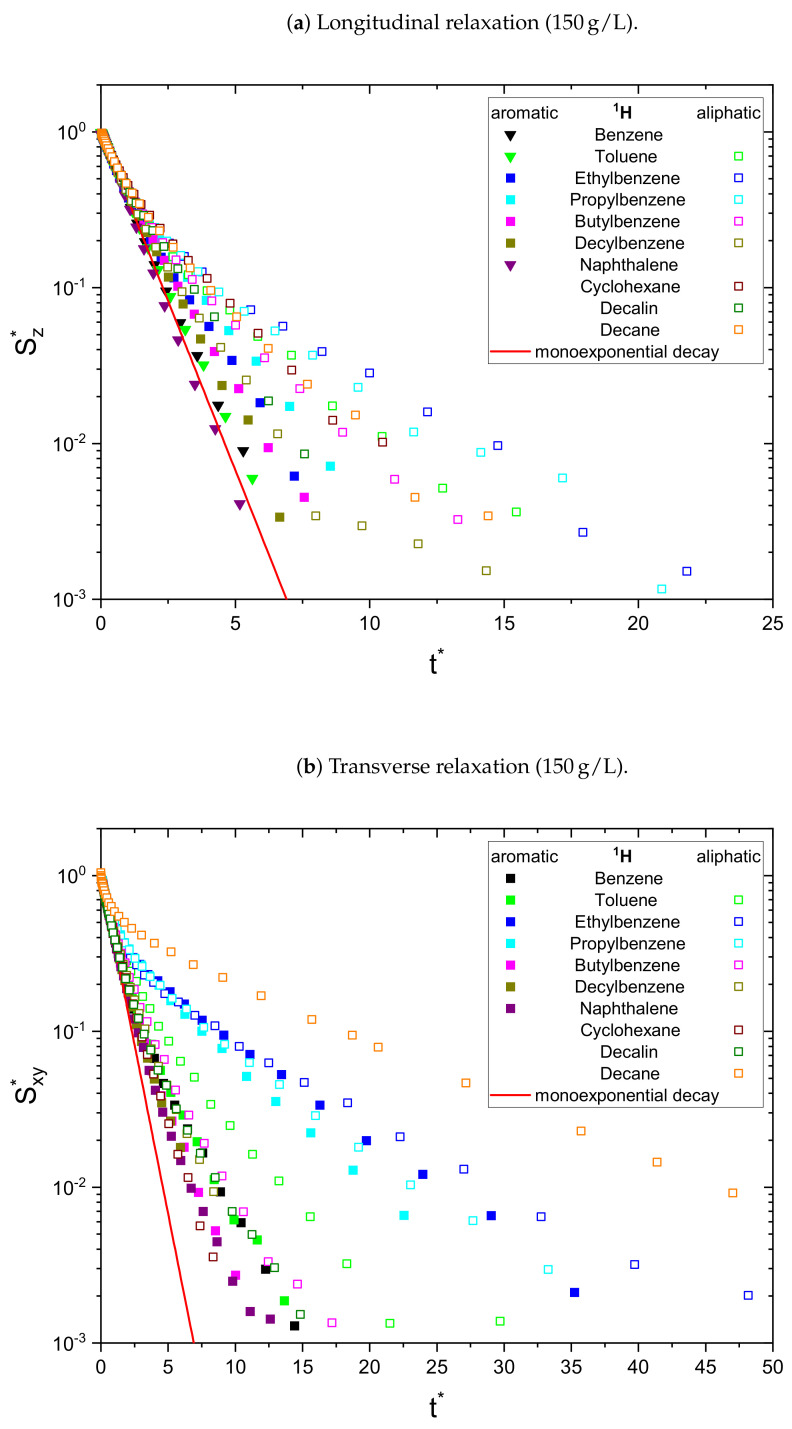
Longitudinal (**a**) and transverse (**b**) relaxation decays of the maltene protons in the asphaltenic solution, consisting of 95 vol% benzene-d_6_ and 5 vol% of the investigated maltene with additional 150 g/L asphaltene. For a qualitative comparison, the time axis is rescaled (see Equation (12)) to reflect multiples of ${\bar{R}}_{1,2}^{-1}$ and the signal is normalized to unity at t* = 0 (see Equations (13) and (14)). The needed parameters were obtained from a monoexponential (∇) or biexponential (☐) fit.

**Figure 3:**
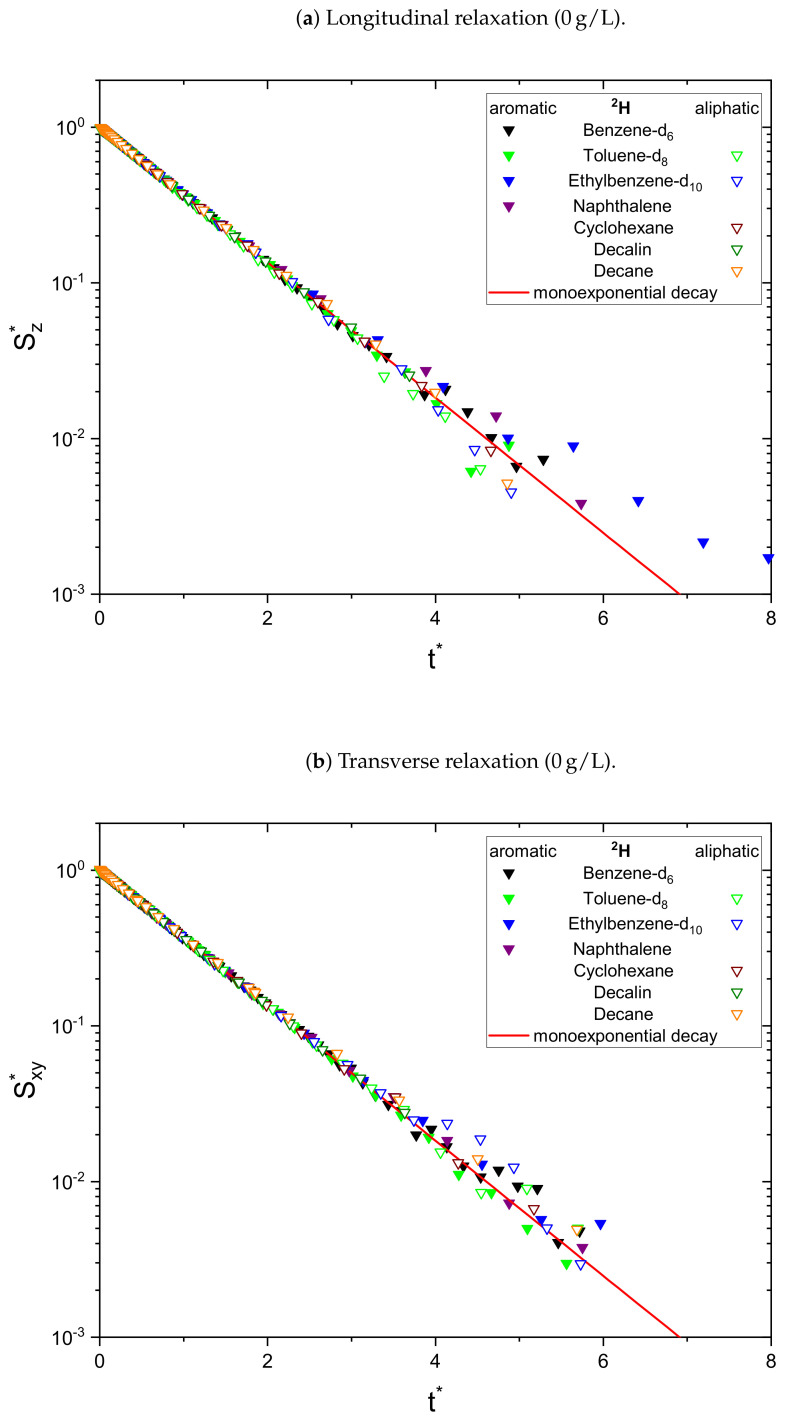
Longitudinal (**a**) and transverse (**b**) relaxation decays of the maltene deuterons in the asphaltene-free solution, consisting of 95 vol% benzene and 5 vol% of the investigated deuterated maltene. For a qualitative comparison, the time axis is rescaled (see Equation (12)) to reflect multiples of ${\bar{R}}_{1,2}^{-1}$ and the signal is normalized to unity at t* = 0 (see Equations (13) and (14)). The needed parameters were obtained from a monoexponential (∇) fit.

**Figure 4:**
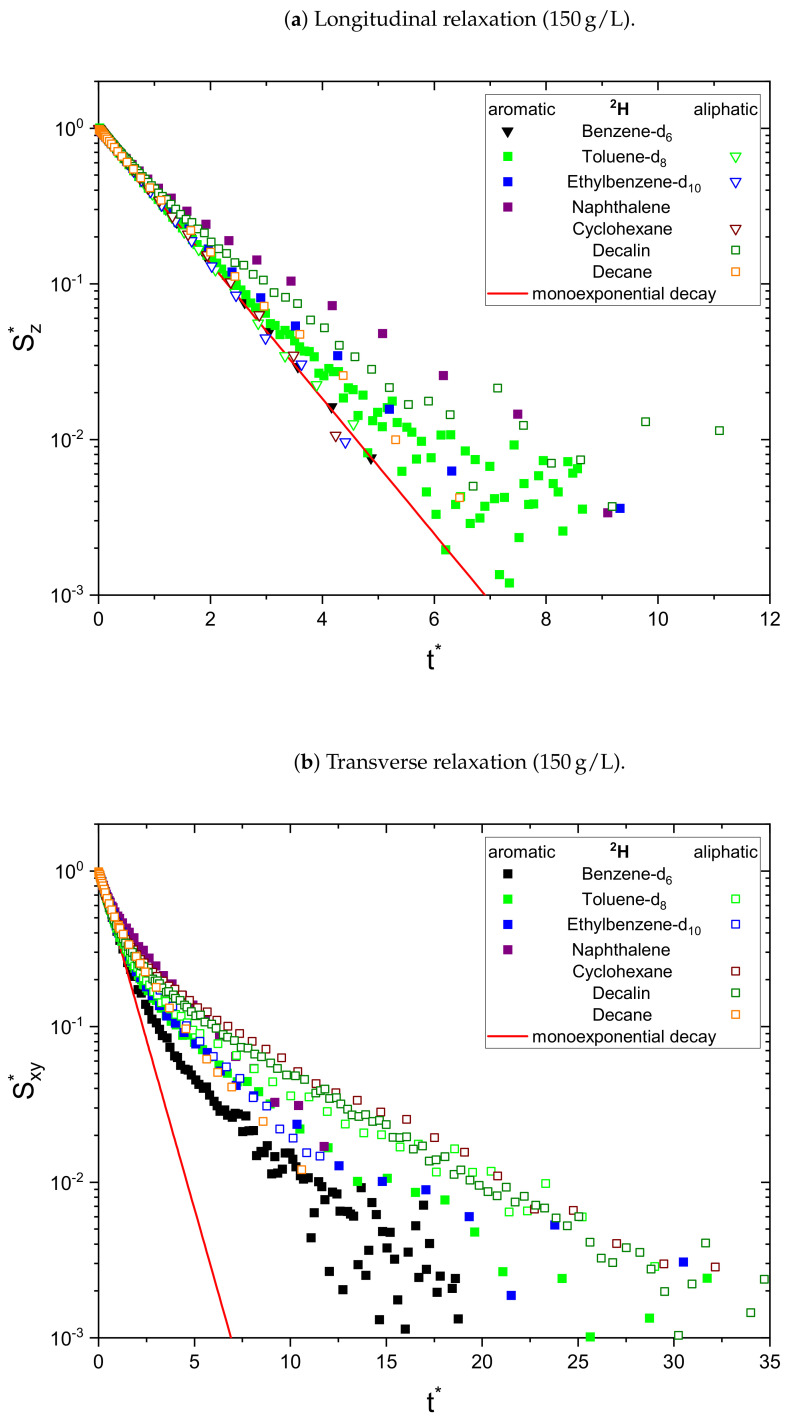
Longitudinal (**a**) and transverse (**b**) relaxation decays of the maltene deuterons in the asphaltenic solution, consisting of 95 vol% benzene and 5 vol% of the investigated deuterated maltene with additional 150 g/L asphaltene. For a qualitative comparison, the time axis is rescaled (see Equation (12)) to reflect multiples of ${\bar{R}}_{1,2}^{-1}$ and the signal is normalized to unity at t* = 0 (see Equations (13) and (14)). The needed parameters were obtained from a monoexponential (∇) or biexponential (☐) fit.

The relaxation decays of the protonic and deuterated maltenes in the asphaltene-free and the asphaltenic solution are displayed in Figure 1 and Figure 2 and Figure 3 and Figure 4, respectively. The different maltenes are distinguished by different colors, while the symbols indicate the chosen fit to obtain the relaxation time constants. A monoexponential decay is indicated by a red line.

In the absence of asphaltene (Figure 1) the relaxation decays of the maltene protons can be described sufficiently well by a monoexponential fit, except for the transverse relaxation of decalin, which can be very well fitted by a sum of two exponential functions using Equation (8). The biexponential relaxation of decalin results from the coexistence of a mixture of cis- and trans-isomers of decalin (see Appendix D). In presence of the asphaltene (Figure 2), most of the relaxation decays become non-monoexponential and are therefore fitted with a sum of two exponential functions. In fact, only the longitudinal relaxation of benzene, toluene, and naphthalene can still be described sufficiently well by a monoexponential decay. Furthermore, the deviation from a monoexponential relaxation is more pronounced for the transverse relaxation than the longitudinal relaxation.

**Figure 5:**
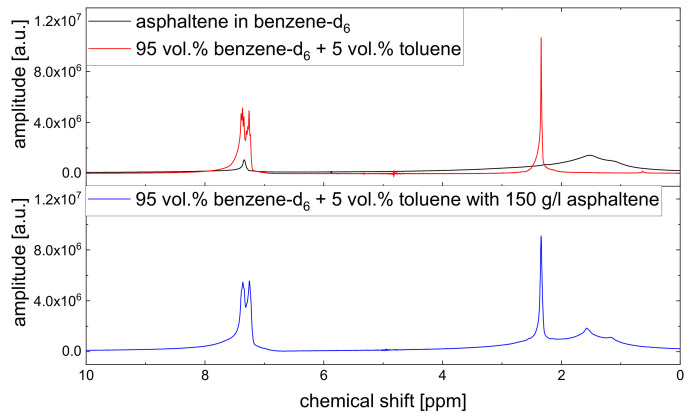
Example for the signal overlap of maltene and asphaltene protons. Top: ${}^{1}$H-NMR spectra of the asphaltene in benzene-d${}_{6}$ and the asphaltene-free toluene solution. Bottom: ${}^{1}$H-NMR spectrum of the asphaltenic toluene solution. The ppm scale of the red and blue spectra is calibrated to the toluene CH_3_ signal at 2.34 ppm. Since the aromatic signal in the black spectrum results not only from the asphaltene, but also from non-deuterated benzene molecules (see Section 4.2), the ppm value of this signal is set to 7.34 ppm. The amplitudes were normalized by the number of scans used to acquire the spectrum.

Since asphaltenes are a proton-rich species consisting of many different molecular structures, the signal of the protons located on the asphaltenes and the maltenes overlap, as can be seen, for example, in Figure 5. Since the time constants characterizing the relaxation of the asphaltene protons are of a comparable order to the relaxation time constants of the maltene protons (Section 4.2), the observed relaxation decays reflect the dynamic of both proton species. Therefore, relaxation experiments with a different nucleus, not abundant in asphaltenes, are necessary to unequivocally link the non-monoexponential relaxation to the maltenes. As one can see in Figure 4, the non-monoexponential character of the relaxation persists for the deuterated maltenes with the similarity that the deviations from a monoexponential decay are more pronounced for the transverse relaxation. The relaxation of the deuterated maltenes in the absence of the asphaltene is well described by a monoexponential fit for all investigated maltenes (see Figure 3).

### 4.2. Relaxation Time Constants

**Figure 6:**
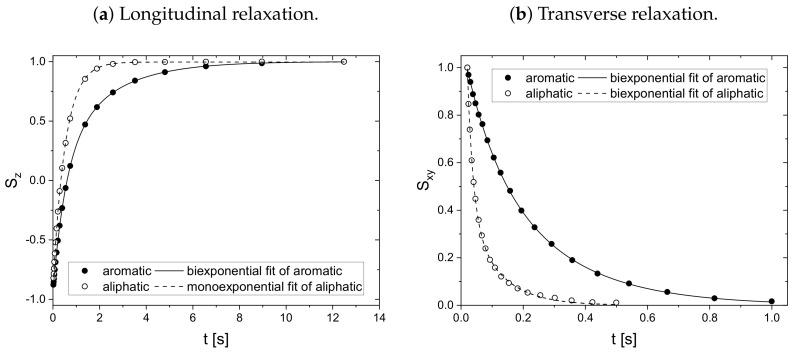
Longitudinal and transverse relaxation curves of the aromatic and aliphatic protons in a solution consisting of 150 g/L asphaltene in benzene-d_6_.

As mentioned before, the relaxation decays of the maltene protons consist of the signals of both the protons located on the maltenes and on the asphaltenes. To access the relaxation of the asphaltene, a sample consisting of 150 g/L asphaltene in benzene-d${}_{6}$ is prepared. For this sample, the longitudinal (a) and transverse (b) relaxation curves of the aromatic and aliphatic protons are displayed in Figure 6. The fit parameter and the relative weight derived according to Equation (11) can be found in Table 1.

Except for the longitudinal relaxation of the aliphatic protons, the proton relaxation is characterized by two time constants. The signal of the aromatic asphaltene protons overlaps with the signal of the remaining non-deuterated benzene molecules. Regarding the deuteration degree of 99.6 atom%, the non-deuterated benzene-d${}_{6}$ protons correspond approximately to about 15% of the total number of aromatic protons in the asphaltene-benzene-d${}_{6}$ solution. The longer relaxation time constant of the aromatic protons can therefore not be fully attributed to the smaller, more mobile non-deuterated benzene molecules. The relaxation time constants of the aromatic protons reflect rather mainly the dynamic of the aromatic asphaltene protons and to a minor but not negligible extent the dynamic of the non-deuterated benzene molecules. In contrast, the relaxation of the aliphatic protons reflects purely the relaxation of aliphatic protons in the asphaltene molecule.

Note the similar time constant components ($T_{1}\approx 550\mathrm{ms}$ and $T_{2}\approx 95\mathrm{ms}$) of the relaxation of the aromatic and aliphatic protons.

#### 4.2.1. Protonic Maltenes

**Figure 7:**
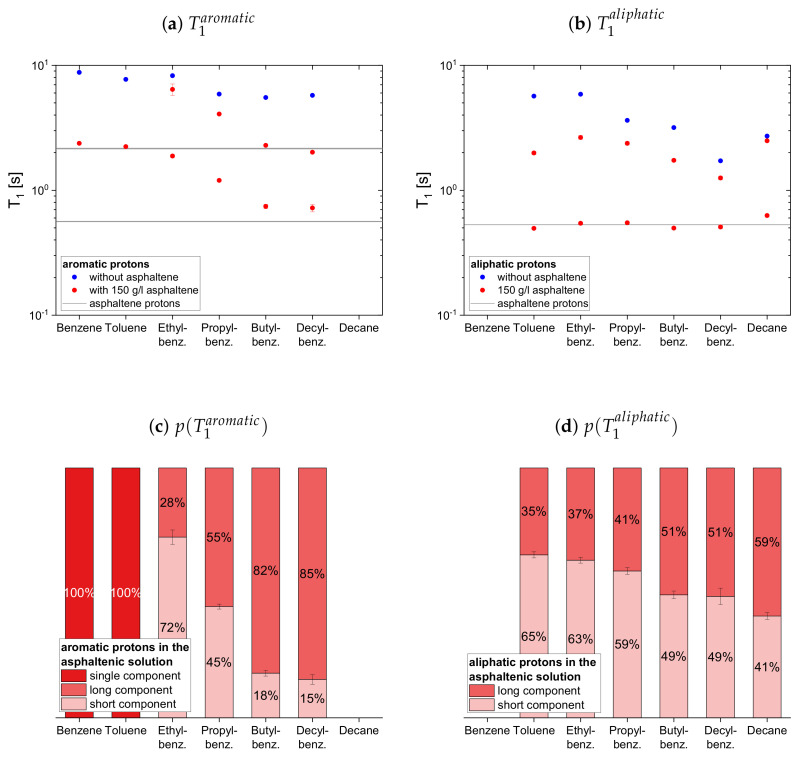
Top: Longitudinal relaxation time constants *T*_1_ of the aromatic (**a**) and aliphatic (**b**) protons in the asphaltene-free and the asphaltenic solution. The black lines indicate the *T*_1_ constants of the asphaltene protons in a benzene-d_6_ solution. Bottom: Relative weight of the *T*_1_ components of the aromatic (**c**) and aliphatic (**d**) protons in the asphaltenic solution. The x-axis distinguishes the different solutions, consisting of 5 vol% of the specified maltene and 95 vol% benzene-d_6_ without or with 150 g/L asphaltene.

**Figure 8:**
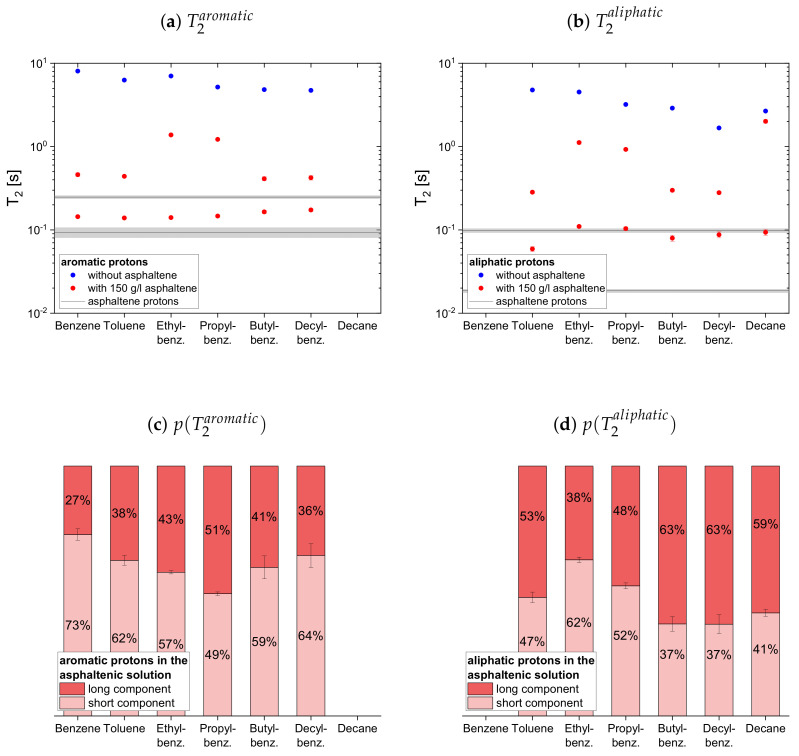
Top: Transverse relaxation time constants *T*_2_ of the aromatic (**a**) and aliphatic (**b**) protons in the asphaltene-free and the asphaltenic solution. The black lines indicate the *T*_2_ constants of the asphaltene protons in a benzene-d_6_ solution. Bottom: Relative weight of the *T*_2_ components of the aromatic (**c**) and aliphatic (**d**) protons in the asphaltenic solution. The x-axis distinguishes the different solutions, consisting of 5 vol% of the specified maltene and 95 vol% benzene-d_6_ without or with 150 g/L asphaltene.

**Figure 9:**
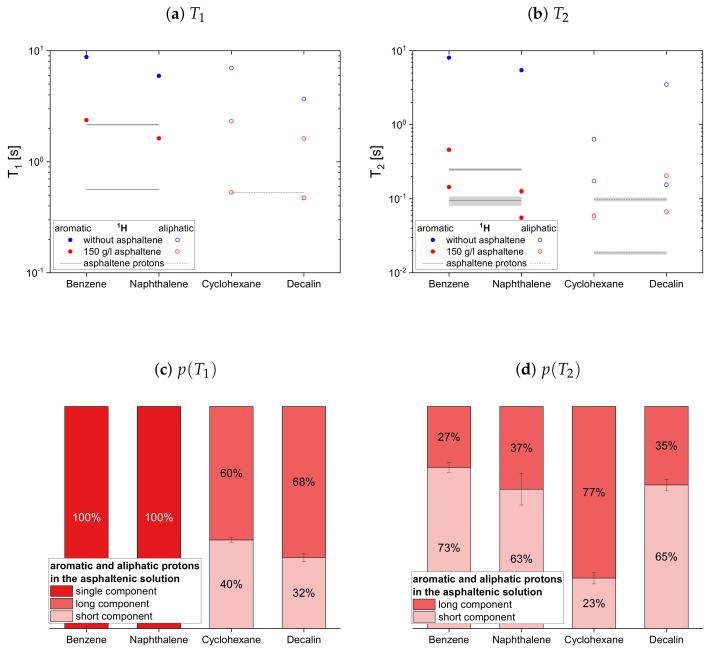
Top: Longitudinal (**a**) and transverse (**b**) relaxation time constants of the aromatic and aliphatic protons of cyclic maltenes in the asphaltene-free and the asphaltenic solution. The black lines indicate the *T*_1_ and *T*_2_ constants of the asphaltene protons in a benzene-d_6_ solution. Bottom: Relative weight of the *T*_1_ (**c**) and *T*_2_ (**d**) components of the maltenes’ protons in the asphaltenic solution. The x-axis distinguishes the different solutions, consisting of 5 vol% of the specified maltene and 95 vol% benzene-d_6_ without or with 150 g/L asphaltene.

In Figure 7, Figure 8 and Figure 9 the relaxation time constants of the maltenes’ proton relaxation are displayed, as well as the relative weight of the two exponential functions used for the biexponential fit of the maltenes’ relaxation in presence of the asphaltene. The relaxation time constants of the protons’ relaxation in the asphaltene-benzene-d${}_{6}$ solution are indicated by a black line, and a gray area indicates the error margins. The maltenes are divided into two subsets differentiating between maltenes with an aliphatic chain and purely cyclic maltenes. Benzene is also included in the first subset as aromatic side-chain free reference maltene.

In absence of the asphaltene, $T_{1}$ and $T_{2}$ are similar for all maltenes except for cyclohexane and decalin. The remarkably short $T_{2}$ of cyclohexane is, as well as the short $T_{2}$ component of decalin, a consequence of an interconversion process present in the cis isomers (see Appendix D). Furthermore, the shorter time constants are found for the larger, less mobile maltenes.

In presence of the asphaltene, two time constants are required to describe the proton relaxation sufficiently, except for the longitudinal relaxation of benzene, toluene, and naphthalene.

Regarding the relaxation of the first subset of maltenes in presence of the asphaltenes, the short $T_{1}$ component of the aliphatic protons, as well as the short $T_{2}$ component of both, aromatic and aliphatic protons, are quite constant to slightly increasing with the length of the side chain. The values of these short components are close to a relaxation time constant found for the asphaltene in benzene-d${}_{6}$. For the short $T_{1}$ component of the aromatic protons, a decrease with the increase of the length of the side chain, from the longer $T_{1}$ to the shorter $T_{1}$ of the asphaltene protons in benzene-d${}_{6}$, is found. The single $T_{1}$ constants of benzene and toluene fit in the trend of the short $T_{1}$ components of the remaining alkylbenzenes’ aromatic protons and differ little from the long asphaltene $T_{1}$.

The long $T_{1}$ component of the aromatic protons decreases similar to the short component, approaching the long $T_{1}$ of the asphaltenes for butyl- and decylbenzene. After the long $T_{1}$ component of the aliphatic protons increases from toluene to ethylbenzene, it decreases with the increase of the length of the side chain.

Similar to the aromatic and aliphatic short $T_{2}$ components, the long $T_{2}$ components are constant for the “short-chained” maltenes benzene and toluene, as well as for the “long-chained” maltenes butyl- and decylbenzene. The long $T_{2}$ components of ethyl- and propylbenzene are also similar, but about three times longer than the long $T_{2}$ components of the other alkylbenzenes’ protons. The long $T_{1}$ and $T_{2}$ component of decane is only slightly shorter than its relaxation time constant in the asphaltene-free solution and significantly larger than the corresponding components of decylbenzene, while the short components are only slightly larger.

The relative weight of the long $T_{1}$ component increases with the increase of the length of the side chain. This is more pronounced for the aromatic protons than for the aliphatic ones, while the relative weight of the $T_{2}$ components is roughly the same.

The relaxation time constants of the second maltene subset, representing aromatic and aliphatic cyclic maltenes, are presented with their relative weights in Figure 9. As mentioned above, only the longitudinal proton relaxation of the aromatic cyclic maltenes is described by a single time constant. Like in the asphaltene-free solution, the shorter time constants are found for the bicyclic maltene, except for the transverse relaxation of the aliphatic cyclic maltenes, where the $T_{2}$ components are similar.

The long $T_{1}$ component of the aliphatic cyclic maltenes is similar to the single $T_{1}$ of the aromatic ones, which are close to the long $T_{1}$ of the aromatic asphaltene protons in benzene-d${}_{6}$, while for the aliphatic cyclic maltenes the short $T_{1}$ component is close to the $T_{1}$ of the aliphatic asphaltene protons. The relative weight of the $T_{1}$ components are similar, with a relative weight of about 65% for the long $T_{1}$ component.

The $T_{2}$ components of the cyclic maltenes are all close to time constants found for the transverse relaxation of aromatic and aliphatic asphaltene protons in benzene-d${}_{6}$. The short $T_{2}$ component is the major component with a relative weight higher than 60% except for cyclohexane, where the long component is the major component.

#### 4.2.2. Deuterated Maltenes

**Figure 10:**
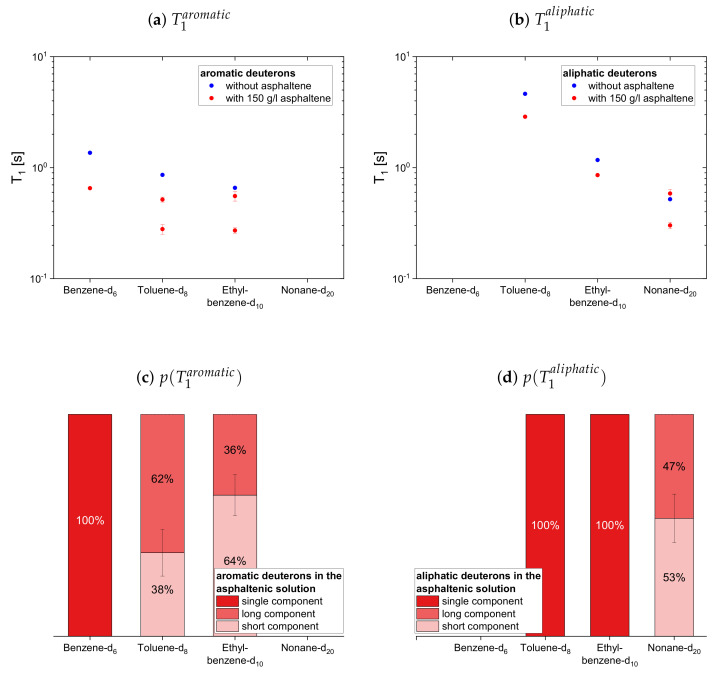
Top: Longitudinal relaxation time constants *T*_1_ of the aromatic (**a**) and aliphatic (**b**) deuterons in the asphaltene-free and the asphaltenic solution. Bottom: Relative weight of the *T*_1_ components of the aromatic (**c**) and aliphatic (**d**) deuterons in the asphaltenic solution. The xaxis distinguishes the different solutions, consisting of 5 vol% of the specified maltene and 95 vol% benzene without or with 150 g/L asphaltene.

**Figure 11:**
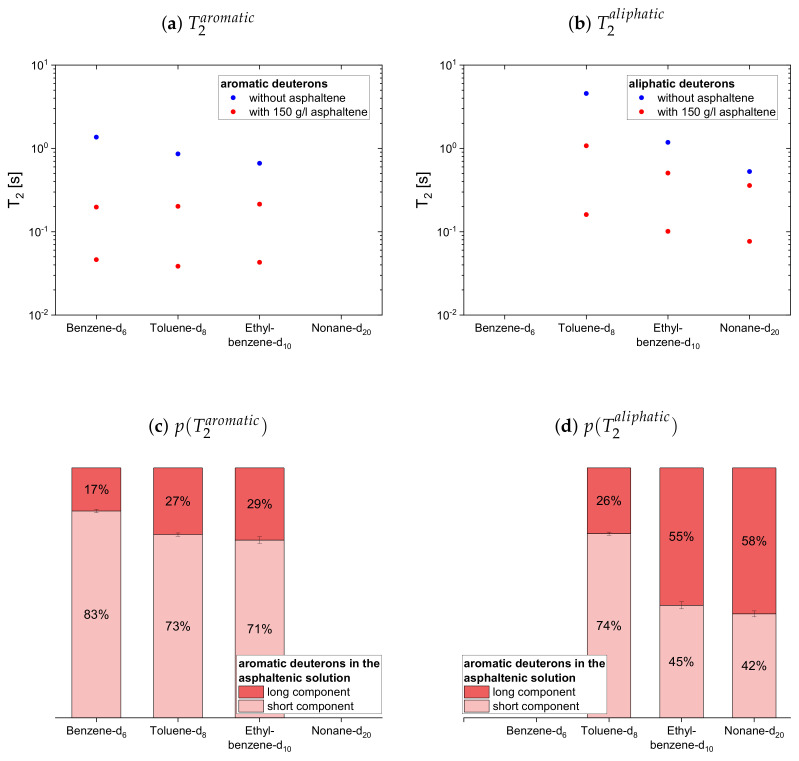
Top: Transverse relaxation time constants *T*_2_ of the aromatic (**a**) and aliphatic (**b**) deuterons in the asphaltene-free and the asphaltenic solution. Bottom: Relative weight of the *T*_2_ components of the aromatic (**c**) and aliphatic (**d**) deuterons in the asphaltenic solution. The x-axis distinguishes the different solutions, consisting of 5 vol% of the specified maltene and 95 vol% benzene without or with 150 g/L asphaltene.

**Figure 12:**
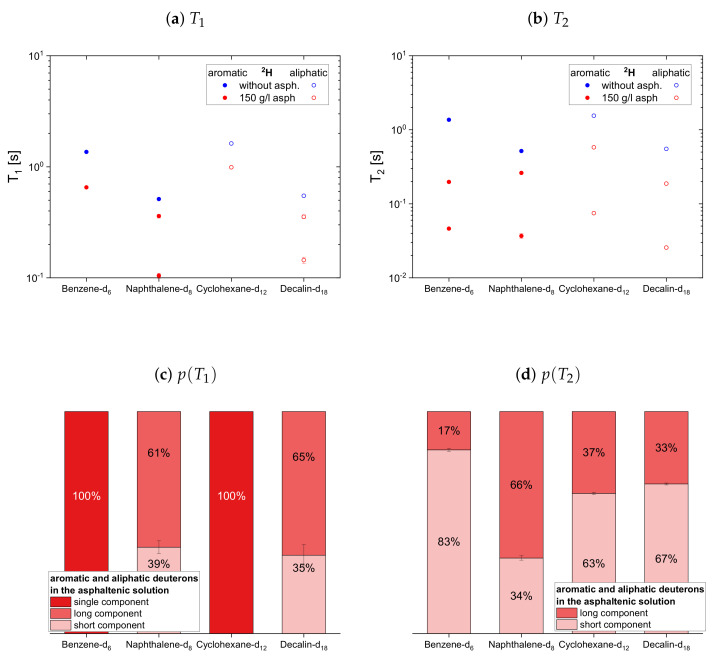
Top: Longitudinal (**a**) and transverse (**b**) relaxation time constants of the aromatic and aliphatic deuterons of cyclic maltenes in the asphaltene-free and the asphaltenic solution. Bottom: Relative weight of the *T*_1_ (**c**) and *T*_2_ (**d**) components of the maltenes’ deuterons in the asphaltenic solution. The x-axis distinguishes the different solutions, consisting of 5 vol% of the specified maltene and 95 vol% benzene without or with 150 g/L asphaltene.

The deuteron relaxation time constants of the deuterated maltenes and their relative weights are displayed in Figure 10, Figure 11 and Figure 12. Similar to the relaxation of the protonic maltenes, the longitudinal deuteron relaxation of some deuterated maltenes in presence of the asphaltene can still be described by a single time constant, while the transverse relaxation is always characterized by two time constants.

The longitudinal and transverse relaxation time constants obtained for the aromatic deuterons in presence of the asphaltene do not differ much between benzene-d${}_{6}$, toluene-d${}_{8}$ and ethylbenzene-d${}_{10}$. In contrast, a decrease of the time constants is observed for the aliphatic deuterons from toluene-d${}_{8}$ via ethylbenzene-d${}_{10}$ to nonane-d${}_{20}$. The relative weights of the two $T_{1}$ components are roughly equal, while for $T_{2}$ often the short component dominates.

In the case of the cyclic maltenes, only the longitudinal deuteron relaxation of benzene-d${}_{6}$ and cyclohexane-d${}_{12}$ can be described by a single $T_{1}$. In the presence and absence of asphaltenes, the shorter time constants are mainly found for the bicyclic maltenes. Merely the long $T_{2}$ component of the aromatic cyclic maltenes does not follow this trend, as the long $T_{2}$ component of benzene-d${}_{6}$ is slightly shorter than the corresponding $T_{2}$ component of naphthalene-d${}_{8}$. Similar to the proton relaxation, the long $T_{1}$ component exhibits a relative weight of roughly 65%, while, except for naphthalene-d${}_{8}$, a higher relative weight is found for the short $T_{2}$ component.

### 4.3. Mean Relaxation Rate Constants

**Figure 13:**
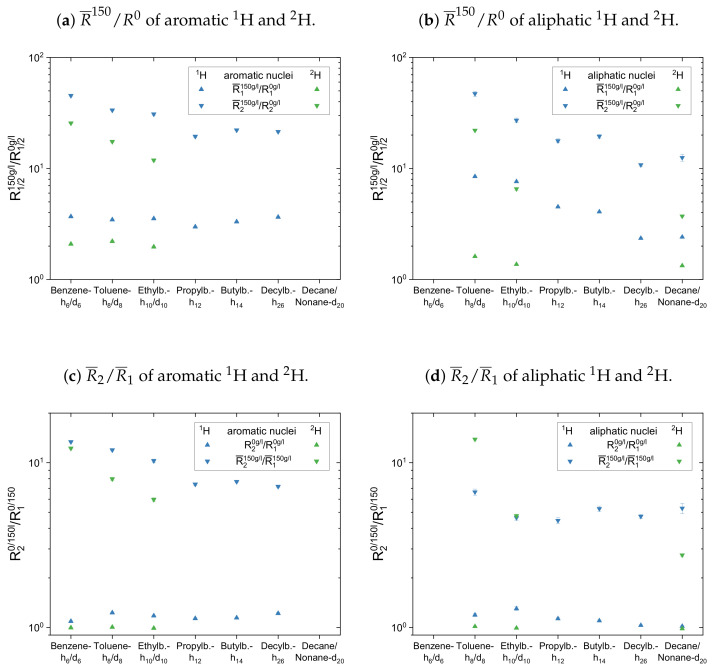
Top: Mean relaxation rates of the aromatic (**a**) and aliphatic (**b**) maltenes’ hydrogen isotopes in the presence of the asphaltene normalized to the corresponding relaxation rates in the asphaltene-free solution. Bottom: ${\bar{R}}_{2}/{\bar{R}}_{1}$ ratio of the investigated aromatic (**c**) and aliphatic (**d**) maltenes’ nuclei in the asphaltenic and asphaltene-free solution.

**Figure 14:**
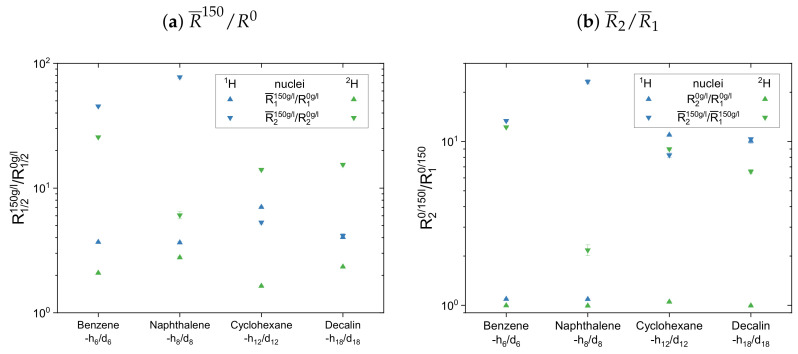
(**a**) Mean relaxation rate constants of the cyclic maltenes’ hydrogen isotopes in the asphaltenic solution normalized by the corresponding relaxation rate constants in the asphaltene-free solution, and (**b**) ${\bar{R}}_{2}/{\bar{R}}_{1}$ ratio of the investigated cyclic maltenes’ nuclei in both solutions.

The calculated mean relaxation rate constants (Equation (10)) for the protonic and the deuterated maltenes can be found in the Appendix C in Table A2 and Table A3, respectively. In Figure 13 and Figure 14, two different normalized mean relaxation rate constants are presented for the two maltene subsets. In the top panel of Figure 13, the maltenes mean relaxation rate constants (${\bar{R}}_{1}$ and ${\bar{R}}_{2}$) in the asphaltenic solution are divided by the corresponding relaxation rate constants in the asphaltene-free solution to visualize the impact of the asphaltene on the maltenes rate constant describing the relaxation. The ${\bar{R}}_{2}/{\bar{R}}_{1}$ ratio of the relaxing nuclei in the different solutions is displayed in the bottom panel of Figure 13. For comparison, the diagrams contain the normalized mean relaxation rate constants of both the protonic and the deuterated maltenes, while aromatic and aliphatic nuclei are presented side by side in different plots.

The maltenes ${\bar{R}}^{150}/R^{0}$ ratios in Figure 13a,b and Figure 14a are always larger than one, i.e., the relaxation of the maltenes is shortened by the presence of asphaltene. The higher ${\bar{R}}^{150}/R^{0}$ ratios can be found for the transverse relaxation, which results in ${\bar{R}}_{2}/{\bar{R}}_{1}$ ratios, for both ${}^{1}$H and ${}^{2}$H, significantly larger than one in the asphaltenic solution. Furthermore, the ${\bar{R}}^{150}/R^{0}$ ratios are higher for the proton relaxation than for the deuteron relaxation. These observations are not true for cyclohexane and decalin in Figure 14 due to their already shortened transverse relaxation in absence of the asphaltene (see Appendix D).

For the first subset, the normalized proton and deuteron mean relaxation rate constants show a similar trend. The ${\bar{R}}^{150}/R^{0}$ ratio of the aromatic hydrogen isotopes is quite constant for the longitudinal relaxation, but shows a decrease from benzene-h${}_{6}$/d${}_{6}$ to ethylbenzene-h${}_{10}$/d${}_{10}$ for the transverse relaxation. With a further increase of the length of the aliphatic side chain, the ${\bar{R}}_{2}^{150}/R_{2}^{0}$ ratio of the aromatic ring protons does not change any further. The aliphatic chain protons, however, show for both, the longitudinal and transverse relaxation a decrease of the ${\bar{R}}^{150}/R^{0}$ ratio with an increase of the side chain length. In principle, this is also seen for the chain deuterons, whereas the decrease is strongly damped in the case of the longitudinal mean relaxation rate constants. Note further, that the decrease of ${\bar{R}}_{2}^{150}/R_{2}^{0}$ from toluene-d${}_{8}$ to ethylbenzene-d${}_{10}$ is more pronounced than in the case of the protonic counterparts.

The ${\bar{R}}_{2}/{\bar{R}}_{1}$ ratios show then a similar trend for the aromatic and aliphatic nuclei. In the asphaltene-free solution, the $R_{2}/R_{1}$ ratio differs only little from unity, slightly more for the protons than for the deuterons. In the asphaltenic solution, the maltene ${\bar{R}}_{2}/{\bar{R}}_{1}$ ratios are noticeably increased, and the highest ratio is found for benzene-h${}_{6}$/d${}_{6}$ and toluene-h${}_{8}$/d${}_{8}$, respectively. Afterward the ${\bar{R}}_{2}/{\bar{R}}_{1}$ ratio decreases for both, aromatic and aliphatic nuclei, until a plateau is reached since the ${\bar{R}}_{2}/{\bar{R}}_{1}$ ratios of butyl- and decylbenzene differ little. Furthermore, the ${\bar{R}}_{2}/{\bar{R}}_{1}$ ratio of decane’s protons fits very well in this plateau. In contrast, a visible difference exists between the${\bar{R}}_{2}/{\bar{R}}_{1}$ ratio of the deuterons of ethylbenzene-d${}_{10}$ and nonane-d${}_{20}$. Furthermore, the decrease of the ${\bar{R}}_{2}/{\bar{R}}_{1}$ ratio between toluene-d${}_{8}$ and ethylbenzene-d${}_{10}$ is more pronounced and their ${\bar{R}}_{2}/{\bar{R}}_{1}$ ratios are, in the first subset, the only ones larger than the ratios of their protonic versions.

In the second subset in Figure 14 the normalized mean relaxation rate constants of the aliphatic cyclic maltene protons differ from the trend seen so far, as the shortening of their transverse relaxation in presence of the asphaltene is, due to their already shortened relaxation in the asphaltene-free solution, similar to the shortening of the longitudinal relaxation. Their ${\bar{R}}_{2}/{\bar{R}}_{1}$ ratio in the asphaltenic solution and the asphaltene-free solution are thus almost identical. Although the comparison with the aromatic cyclic protonic maltenes is therefore limited, the ${\bar{R}}_{2}/{\bar{R}}_{1}$ ratios of cyclohexane and decalin in the asphaltenic solution are of comparable size, as the corresponding ratio of benzene. Furthermore, are the ${\bar{R}}_{2}/{\bar{R}}_{1}$ ratios of the deuterated cyclic aliphatic maltenes comparable/slightly smaller than the ratios of the protonic counterparts, which is in agreement with the overall relation of proton and deuteron ${\bar{R}}_{2}/{\bar{R}}_{1}$ observed for the first subset.

From this relation differs the ${\bar{R}}_{2}/{\bar{R}}_{1}$ ratios of naphthalene and naphthalene-d${}_{8}$, with a deuteron ${\bar{R}}_{2}/{\bar{R}}_{1}$ ratio more than ten times smaller than the proton ratio. This results from a significantly lower shortening of the transverse relaxation of naphthalene-d${}_{8}$ compared with naphthalene, while the shortening of the longitudinal relaxation is similar. As a result, the highest and lowest ${\bar{R}}_{2}/{\bar{R}}_{1}$ ratio of all maltenes is found for naphthalene and naphthalene-d${}_{8}$, respectively.

Therefore, it is difficult to identify a trend for the influence of the asphaltene presence on the cyclic maltenes. Regarding their size, the higher proton ${\bar{R}}_{2}/{\bar{R}}_{1}$ ratio is found for the bicyclic maltenes, while in the case of the deuterated maltenes the bicyclic maltenes exhibit the lower ${\bar{R}}_{2}/{\bar{R}}_{1}$ ratio compared to the monocyclic maltenes. In terms of aromaticity, a slightly higher ${\bar{R}}_{2}/{\bar{R}}_{1}$ ratio is found for benzene-h${}_{6}$/d${}_{6}$ compared to the aliphatic cyclic maltenes (${}^{1}$H and ${}^{2}$H). However, the ratio of naphthalene-h${}_{8}$/d${}_{8}$ is, as mentioned before, significantly higher (${}^{1}$H) or lower (${}^{2}$H) than the ratios of the remaining cyclic maltene.

### 4.4. Diffusion

**Figure 15:**
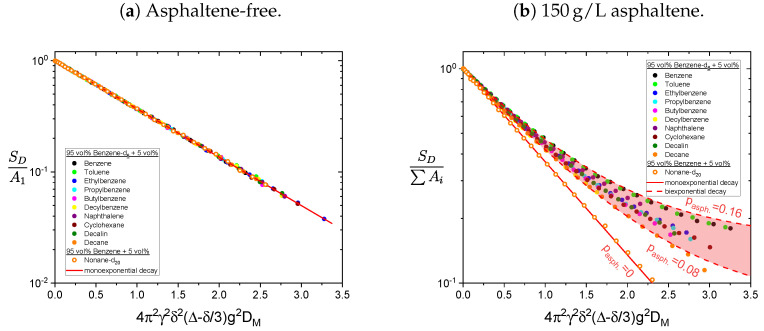
PGSTE signal decay of the protonic maltenes and nonane-d20 in the (**a**) asphaltene-free and the (**b**) asphaltenic solution. The solid line represents a monoexponential decay, while the area between the dashed lines corresponds to biexponential decays with different relative contributions *p*_asph._ of the asphaltene to the decay. The diffusion coefficient of the asphaltene was fixed at *D*_asph._ = 3.6 × 10^−11^ m^2^/s. To obtain the signal decay of nonane-d20 the gradient strength was varied up to 5 T/m.

**Figure 16:**
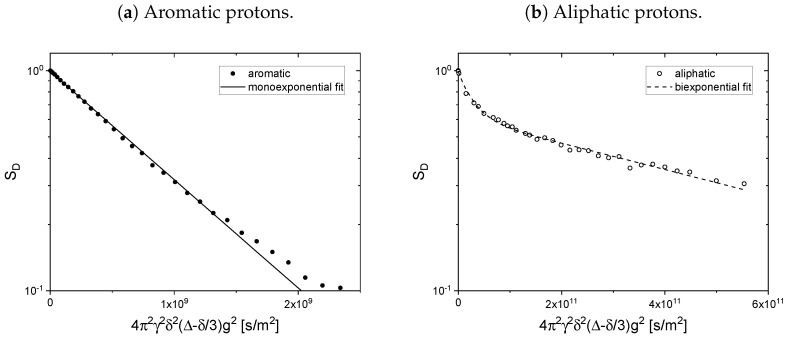
PGSTE signal decay of the aromatic (**a**) and aliphatic (**b**) protons in a solution of 150 g/L asphaltene in benzene-d_6_. To obtain the signal decay of the aliphatic protons, the gradient strength was varied up to 10 T/m.

The, compared to the pulse spacing in the PGSTE sequence, long relaxation time constants of the asphaltene protons (see Table 1) cause only a small attenuation of the asphaltene signal during the diffusion measurement. Therefore, the contribution of the asphaltene protons to the obtained PGSTE signal cannot be neglected in determining the diffusion coefficients of the maltenes in the asphaltenic solution. Accordingly, the obtained proton PGSTE decays differ from a monoexponential decay in the asphaltenic solution (see Figure 15). However, the variation of the gradient strength was adjusted to obtain the diffusion coefficients of the maltenes and is therefore not sufficient to reliably fit a sum of two exponential functions to additionally extract the asphaltene’s diffusion coefficient. The asphaltene’s diffusion coefficient was therefore determined in the solution of 150 g/L asphaltene in benzene-d${}_{6}$ and the obtained signal is displayed in Figure 16 for the aromatic (a) and the aliphatic (b) protons.

As already seen in the determination of the relaxation time constants (see Figure 6), the signal overlap of aromatic asphaltene protons and protons of remaining non-deuterated benzene molecules results in a superposition of the individual signal decays. By comparing the PGSTE signal decay of the aromatic and aliphatic protons in Figure 16, one notices the significantly faster decay of the aromatic protons signal. Since asphaltenes are, due to their definition, a heterogeneous class of molecules, a distribution of diffusion coefficients is not a surprising observation. However, considering the molecular weight range of asphaltenes as the heaviest part of crude oil, a diffusion coefficient of the order of $10^{-9}$ m^2^/s, as obtained by a single-exponential fit of the aromatic protons signal decay (see Table 2), is not expected and can therefore be mainly attributed to the non-deuterated benzene molecules with high probability (For comparison, the self-diffusion coefficient of the remaining non-deuterated benzene molecules in a sample consisting of 100% benzene-d${}_{6}$ was determined to $(1.842\pm 000.7)\times 10^{-9}$ m^2^/s). The asphaltene diffusion coefficients are therefore determined from the signal decay of the aliphatic protons, whose signal does not overlap with the signal of non-deuterated solvent molecules. The signal decay could be fitted sufficiently well by a sum of two exponential functions (see Table 2) revealing diffusion coefficients of $(3.6\pm 0.3)\times 10^{-11}$ m^2^/s and $(1.38\pm 0.07)\times 10^{-12}$ m^2^/s, respectively. These values seem reasonable in comparison with the self-diffusion coefficients of about $1\times 10^{-10}$ m^2^/s found for asphaltenes in solutions at concentrations not higher than 30 g/L [50,51,52].

For the gradient strengths used to determine the protonic maltenes diffusion coefficients in the asphaltenic solution, only the faster diffusion of the asphaltene has to be taken into account. The signal decays of the maltenes in the asphaltenic solution are therefore fitted with a biexponential fit according to Equation (9), with a fixed second diffusion coefficient $D_{2}=3.6\times 10^{-11}$ m^2^/s.

The signal decays of the maltenes in the absence and presence of the asphaltene are displayed in Figure 15. For comparison, the signal decays are normalized like the relaxation decays in Section 4.1, i.e., the signal is normalized to the sum of the two exponential functions amplitude and the x-axis is rescaled with the obtained maltene diffusion coefficient $D_{M}$.

In absence of the asphaltene, the decay is perfectly described by a single-exponential function, whereas in presence of the asphaltene the additional diffusion of the asphaltenes is seen in a deviation from a monoexponential decay. The relative contribution of the asphaltene to the fit $p_{\mathrm{asph}.}$ is calculated according to Equation (11) and varies between 8% and 16%. The monoexponential decay of the deuteron signal of nonane-d${}_{20}$ in the presence of the asphaltene supports the assumption that the deviation from a monoexponential decay of the protonic maltenes reflects the diffusion of the asphaltene rather than representing different diffusion coefficients of the maltenes.

The diffusion coefficients of the maltenes are listed in Table 3 together with the $D^{150g/l}/D^{0g/l}$ ratio. The ratio reflects to what extent the diffusion coefficient, and thus the translational motion of the maltenes, is diminished by the presence of the asphaltene.

The diffusion coefficients of the maltenes were mostly decreased by roughly 30% to 35% in the presence of the asphaltenes. With the minimal decrease of 20%, decane differs the most and is the maltene with the least impairment of its translational motion. This decrease is notably weaker than the similar long aliphatic chain molecule nonane-d${}_{20}$ and the phenyl ring bearing decylbenzene, which is the most restricted alkylbenzene. With a decreasing side chain length, the ratio increases to a maximum for toluene, which reflects its smaller size. The cyclic hydrocarbons are similarly affected by the presence of the asphaltene, whereby the difference between the aromatic ring molecules is slightly bigger than between the aliphatic ones. However, the overall differences between the maltenes are rather small, and no pronounced effect of the asphaltene on the translational motion of any of these molecules is observed.

## 5. Discussion

In the presence of 150 g/L asphaltene, the longitudinal and transverse relaxation of most investigated maltenes are no longer monoexponential and can be sufficiently described by a fit of two exponential functions. For the ${}^{1}$H measurements, the signal of the asphaltene and maltene protons overlap, and the obtained relaxation decays reflect not only the motion of the maltenes, but also of the asphaltene. From the two time constants, the shorter is often similar to the relaxation time constants found for asphaltene in benzene-d${}_{6}$. However, the non-monoexponential, biexponentially fittable, relaxation decays are not only observed for the protonic maltene solutions, but also for the ${}^{2}$H relaxation of their deuterated analogs, where the asphaltenes are not visible and the non-monoexponential decays purely reflect the maltenes dynamic.

In the current picture of the maltene–asphaltene interaction, the two relaxation time constants observed in the asphaltenic solutions could represent different relaxation environments of the maltenes, which are caused by the proximity of the maltenes to the asphaltenes. The dynamic of maltenes in the intermediate space between the asphaltene clusters is mainly affected by the viscosity of the solution, but not the direct interaction with the asphaltene structures. The longer relaxation time constant could therefore be attributed to these bulk molecules. In contrast, the molecules in close proximity to the asphaltene macroaggregates experience an additional relaxation mechanism due to paramagnetic species VO^2+^ in the asphaltenes, and a stronger restriction of their motion, due to steric hindrances or additional interactions such as π-stacking. These molecules then exhibit a shorter relaxation time than the bulk molecules farther apart from the asphaltene clusters. The border between these two environments is not expected to be sharp, but rather a more or less uniform transition, so that a distribution of relaxation times could be expected.

For further comparison, the mean relaxation rate constants are constructed from the $T_{1,2}$ components and the corresponding relative weights. The ${\bar{R}}_{1,2}$ condense the relaxation of the investigated nuclei into a single parameter and ease the general comparison of the different maltene solutions. In presence of the asphaltene, the relaxations of the investigated nuclei become faster. Thereby, the transverse relaxation is more affected than the longitudinal relaxation, which was already observed before for a mixture of maltenes with a varying asphaltene concentration [19]. As mentioned above, the shortening of the maltene’s relaxation can be expected due to the presence of paramagnetic species and a slowed maltene’s motion, resulting from the contact and entanglement between the maltenes and the asphaltene macrostructures. The, compared to $R_{1}$, stronger increase of $R_{2}$ indicates the presence of motions with correlation times comparable or slower than the inverse of the Larmor frequency, and thus the latter effect is significant since the presence of paramagnetic species influences the longitudinal and transverse relaxation equally. As a consequence, the ${\bar{R}}_{2}/{\bar{R}}_{1}$ ratios, which reflect the strength of the impairment, are significantly larger than unity in the asphaltenic solution, while in the asphaltene-free solution ${\bar{R}}_{2}/{\bar{R}}_{1}\approx 1$ indicates the extreme narrowing limit.

As a result of an internal motion of cyclohexane and decalin (see Appendix D), the ${\bar{R}}_{2}/{\bar{R}}_{1}$ ratios in the asphaltene-free solution are larger than unity and differ little from the ratios in the asphaltenic solution. The comparability with the other maltenes is therefore limited, since the impact of the asphaltene presence on the dynamic is not clear. Furthermore, the theory and models developed so far have assumed rigid maltenes without considering internal motions, especially not ring flips, which can result in totally different behavior. This then needs to be considered in future oil studies, in case a significant proportion of the maltenes in crude oil exhibit similar internal motions.

For the deuterated maltenes the shortening of $T_{2}$ due to the internal motion is negligible and ${\bar{R}}_{2}/{\bar{R}}_{1}$ ratios close to and significantly larger than unity are observed in the asphaltene-free and the asphaltenic solution, respectively. Comparing the bicyclic ring system with the monocyclic, the lower ${\bar{R}}_{2}/{\bar{R}}_{1}$ ratio is found for the bicyclic one. However, the decrease from the monocyclic to the bicyclic maltene is much stronger for the aromatic ring systems than for the aliphatic ones. In contrast, the ratio of the protonic aromatic bicyclic maltene (naphthalene) is larger than the ratio of benzene. As a result, the ${\bar{R}}_{2}/{\bar{R}}_{1}$ ratios of benzene and benzene-d${}_{6}$ differ little, while for naphthalene and naphthalene-d${}_{8}$, the ratios differ the most among all investigated maltenes. However, the results from the protonic and the deuterated maltene solutions should be compared with caution, since one compares the maltene–asphaltene dynamic (${}^{1}$H) with the maltene dynamic (${}^{2}$H) and in addition different relaxation mechanisms. While the ${}^{1}$H relaxation is caused by the rotational and translational motion of the molecule (as well as the presence of free electrons), the ${}^{2}$H relaxation results mainly from the rotational motion of the molecule (and the presence of free electrons).

Regarding the influence of the side chain length of the maltene, the observed ${\bar{R}}_{2}/{\bar{R}}_{1}$ ratio decreases with the increase of the side chain length, probably approaching a plateau for long side chains, since the ratios of butylbenzene (#C${}_{\mathrm{aliph}.}=4$) and decylbenzene (#C${}_{\mathrm{aliph}.}=10$) differ little. The ratios of decane and decylbenzene are similar, indicating that the phenyl ring is of minor importance for the restrictions the aliphatic chain experiences in presence of the asphaltene. The initial decrease of the ${\bar{R}}_{2}/{\bar{R}}_{1}$ ratio is also observed for the deuterated variants.

However, the ${\bar{R}}_{2}/{\bar{R}}_{1}$ ratios of ethyl- (aliphatic) and propylbenzene (aromatic and aliphatic) are slightly lower than approximated from the trend, which reflects their significantly different long relaxation component, compared to the rest of the maltenes. This is especially well pronounced for the long $T_{2}$ component, as it was shown in Figure 8. Due to the lack of solutions with deuterated analogs, it cannot be verified whether this is a real characteristic of the maltene dynamic.

The self-diffusion coefficients of the maltenes (including nonane-d${}_{20}$) in the high (150 g/L) concentrated asphaltenic solution are only reduced by 30% to 35% compared to their self-diffusion coefficients in the asphaltene-free solution. Merely decane deviates slightly from this observation since its self-diffusion coefficient is reduced by about 20%. Overall, the diffusion of the different maltenes is similar affected by the asphaltene’s presence, and especially no pronounced effect is found for ethyl- and propylbenzene. The impairment of the translational motion is therefore caused by the increased viscosity of the solution due to the high asphaltene concentration. If there is a prolonged interaction time between the maltenes and asphaltenes, it is still short compared to the observed time interval in the diffusion measurements.

## 6. Conclusions

In this work, the relaxation and diffusion of different maltenes in the presence and absence of asphaltene were investigated. The self-diffusion is equally restricted for all maltenes in presence of the asphaltene, due to the increased viscosity of the solution. In contrast to crude oil, the relaxation time constants of asphaltene in solution are considerably long and as a consequence, the ${}^{1}$H relaxation decays in the maltene–asphaltene solution reflect the dynamic of both maltenes and asphaltenes.

In presence of the asphaltene, the maltenes’ relaxation is enhanced, due to the influence of paramagnetic species, as well as the slowdown of the maltenes’ motion upon contact and entanglement with the asphaltene structures. Since $R_{2}$ is more affected than $R_{1}$, the slowdown is of significant importance. The increase of $R_{2}$ and $R_{1}$ due to the presence of asphaltene is more pronounced for ${}^{1}$H in comparison to ${}^{2}$H, but amounts only to an increase of typical 50%. If dipolar interaction were the sole origin of relaxation, this factor should be equivalent to the square of the ratio of the gyromagnetic constant, about 42. If intramolecular contributions were the sole origin, the ratio of relaxation rate constants with and without asphaltene should be identical for ${}^{2}$H and ${}^{1}$H. It is therefore reasonable to assume that relaxation of ${}^{1}$H with the unpaired electrons of the radicals contained in the asphaltenes does contribute to ${}^{1}$H relaxation, but is not dominating.

Overall, a higher ratio of mean relaxation rate constants is often found for the “more aromatic” maltene, i.e., the shorter chained alkylbenzene or the aromatic ring systems, indicating a possible influence of aromatic maltene–asphaltene interactions, e.g., π-stacking. Since the self-diffusion coefficients of the maltenes are all similar, a possible prolonged maltene–asphaltene interaction time due to aromatic interactions is still short compared to the studied diffusion time interval. However, a definite statement about the role of aromatic interactions cannot be made, as the remarkably small mean relaxation rate constants ratio of naphthalene-d${}_{8}$ shows. Hence, more detailed studies are needed.

Most maltenes show at least two distinct relaxation time constants due to internal motions of different parts, e.g., ring and chain. In presence of the asphaltene, the relaxation further becomes non-monoexponential, especially the transverse relaxation. The individual relaxations and the averaged values $R_{2}$ and $R_{1}$ differ from one maltene to another. For some maltenes, the relaxation can be influenced significantly by conformational interconversion processes. Therefore, the distribution of either $T_{1}$ or $T_{2}$, or both (in 2D experiments) of crude oil is much more complicated than conventionally assumed, and is strongly dependent on the composition of the maltenes.

This approach serves as a further step to analyze these compositions from a thorough analysis of the data, especially in two-dimensional experiments. Further modeling and density functions theory computations will help to elucidate the picture, at the same time integrating a realistic model for asphaltene aggregate structure and radical location.

## Figures and Tables

**Table 1:** Fit parameters used to fit the in Figure 6 shown relaxation curves of protons in a solution consisting of 150 g/L asphaltene in benzene-d${}_{6}$, using Equations (13) and (14).

Longitudinal Relaxation			Transverse Relaxation		
Parameter	Aromatic	Aliphatic	Parameter	Aromatic	Aliphatic
$S_{0}$	1.001(2)	0.997(2)	$S_{0}$	0	0
$A_{1}$	−1.11(2)	−1.890(4)	$A_{1}$	0.34(8)	1.80(6)
$A_{2}$	−0.82(2)		$A_{2}$	0.79(8)	0.48(4)
$T_{1_{1}}$ [s]	0.562(7)	0.529(3)	$T_{2_{1}}$ [s]	0.09(2)	0.018(1)
$T_{1_{2}}$ [s]	2.15(4)		$T_{2_{2}}$ [s]	0.25(2)	0.098(6)
$p_{1}$ [%]	57.4(9)		$p_{1}$ [%]	30(7)	79(2)
$p_{2}$ [%]	42.6(9)		$p_{2}$ [%]	70(7)	21(2)

**Table 2:** Fit parameters used to fit the in Figure 16 shown PGSTE signal decays of aromatic and aliphatic protons in a solution of 150 g/L asphaltene in benzene-d${}_{6}$, using Equation (9).

Diffusion			
Parameter	Aromatic	Aliphatic	
$A_{1}$	0.993 ± 0.005	0.375 ± 0.012	38%
$D_{1}$ [m${}^{2}$/s]	$(1.13\pm 0.02)\times 10^{-9}$	$(3.6\pm 0.3)\times 10^{-11}$	
$A_{2}$		0.617 ± 0.011	62%
$D_{2}$ [m${}^{2}$/s]		$(1.38\pm 0.07)\times 10^{-12}$	

**Table 3:** Diffusion coefficients of the maltenes in absence and presence of the asphaltene. The $D^{150g/l}/D^{0g/l}$ ratio reflects the impairment of the translational motion of the maltenes due to the presence of the asphaltene.

Maltene	$D^{0g/l}$ [$10^{-9}$ m${}^{2}$/s]	$D^{150g/l}$ [$10^{-9}$ m${}^{2}$/s]	$\frac{D^{150g/l}}{D^{0g/l}}$
Decalin	1.393 ± 0.005	0.949 ± 0.007	0.681± 0.008
Cyclohexane	1.767 ± 0.007	1.182 ± 0.006	0.669 ± 0.006
Naphthalene	1.409 ± 0.005	0.903 ± 0.007	0.641 ± 0.007
Benzene	1.856 ± 0.006	1.280 ± 0.007	0.690 ± 0.006
Toluene	1.769 ± 0.008	1.256 ± 0.005	0.710 ± 0.006
Ethylbenzene	1.652 ± 0.005	1.162 ± 0.007	0.704 ± 0.006
Propylbenzene	1.511 ± 0.004	1.049 ± 0.006	0.695 ± 0.006
Butylbenzene	1.410 ± 0.005	0.956 ± 0.008	0.678 ± 0.008
Decylbenzene	0.983 ± 0.003	0.646 ± 0.005	0.657 ± 0.006
Decane	1.465 ± 0.004	1.152 ± 0.011	0.786 ± 0.009
Nonane-d${}_{20}$	1.604 ± 0.004	1.140 ± 0.005	0.711 ± 0.005

**Figure A1:**
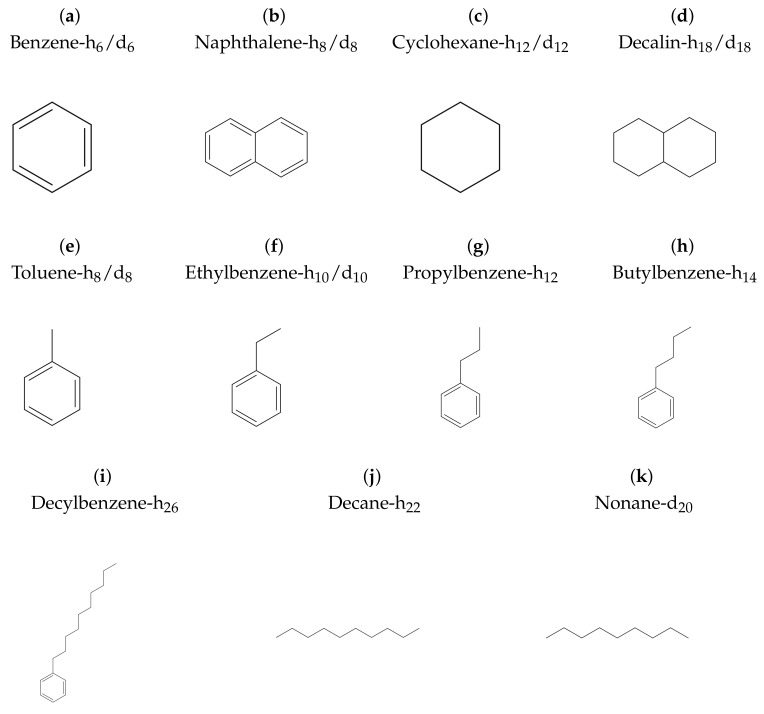
The chemicals used in this study include cycloalkanes (**a**–**d**), alkylbenzenes (**e**–**i**), as well as linear alkanes (**j**,**k**). The cycloalkanes can be distinguished in monocyclic (**a**,**c**) and bicyclic (**b**,**d**), as well as in aromatic (**a**,**b**) and aliphatic (**c**,**d**) ring systems. Protons and deuterons are not shown for clarity. The individual headings specify the used variants (^1^H, ^2^H). MolView was used to generate the structural formulas.

**Figure A2:**
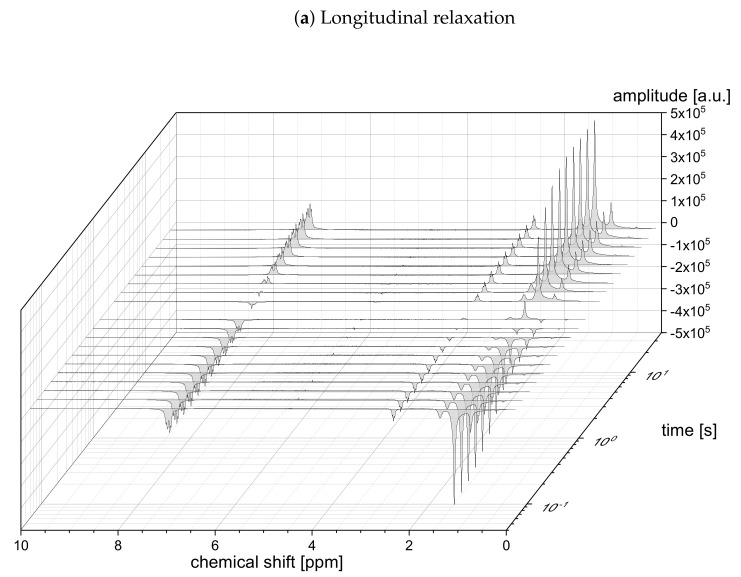

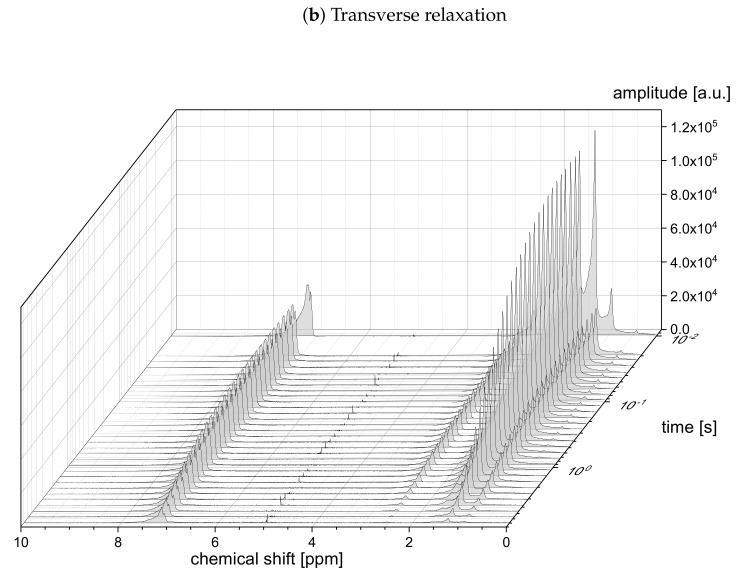
Longitudinal (**a**) and transverse (**b**) relaxation of decylbenzene in the asphaltene-free solution. The chemical shift axis is normalized to the dominant peak in the spectrum at 1.26 ppm, which results from the seven CH_2_ groups, most apart from the phenyl ring. Due to the spectral resolution the signal from the aliphatic chain (0 ppm–3 ppm) and from the phenyl ring (6 ppm–8 ppm) are well distinguishable and the relaxation of each part can be evaluated separately.

## Data Availability

The data presented in this study are openly available in FigShare at [10.6084/m9.figshare.15082656].

## Abbreviations

The following abbreviations are used in this manuscript:

NMR	nuclear magnetic resonance
EPR	electron paramagnetic resonance
IR	inversion recovery
CPMG	Carr-Purcell-Meiboom-Gill

## Appendix A. List of Chemicals and Suppliers

**Table A1:** List of chemicals and suppliers.

Provider	Chemical
Alfa Aesar	Decalin
Merck KGaA	Decane
Fluka Analytical	Ethylbenzene, Naphthalene
Sigma-Aldrich	Benzene, Propylbenzene, Butylbenzene, Decylbenzene, Ethylbenzene-d${}_{10}$, Naphthalene-d${}_{8}$
Carl Roth GmbH + Co. KG	Toluene, Cyclohexane
Cambridge Isotope Laboratories, Inc.	Benzene-d${}_{6}$, Cyclohexane-d${}_{12}$
Chemotrade Chemiehandelsgesellschaft Leipzig mbH & Co. KG	Cyclohexane-d${}_{12}$, Toluene-d${}_{8}$
Deutero GmbH	Decalin-d${}_{18}$
Akademie der Wissenschaften der DDR, Zentralinstitut für Isotopen- und Strahlenforschung	Nonane-d${}_{20}$

## Appendix C. Mean Relaxation Rate Constants (^1^H and ^2^H)

**Table A2:** (Mean) relaxation rate constants of the longitudinal ($R_{1}$) and transverse ($R_{2}$) relaxation of the protonic maltenes in absence and presence of the asphaltene.

Protonic Maltene	Without Asphaltene		With 150 g/L Asphaltene	
	$R_{1}$ [$s^{-1}$]	$R_{2}$ [$s^{-1}$]	${\bar{R}}_{1}$ [$s^{-1}$]	${\bar{R}}_{2}$ [$s^{-1}$]
Decalin	0.272(2)	2.74(8)	1.10(1)	11.4(2)
Cyclohexane	0.1437(4)	1.576(8)	1.012(9)	8.4(2)
Naphthalene	0.1686(5)	0.184(1)	0.616(9)	14.3(3)
Benzene	0.1140(3)	0.1243(8)	0.421(2)	5.64(5)
Toluene (ring)	0.1296(4)	0.156(2)	0.448(3)	5.35(5)
Toluene (CH_3_)	0.176(1)	0.210(4)	1.492(9)	9.9(4)
Ethylbenzene (ring)	0.1210(3)	0.142(1)	0.428(4)	4.40(7)
Ethylbenzene (chain)	0.1704(7)	0.222(5)	1.30(2)	6.0(2)
Propylbenzene (ring)	0.1702(5)	0.193(1)	0.508(2)	3.76(7)
Propylbenzene (chain)	0.276(2)	0.312(4)	1.24(2)	5.5(2)
Butylbenzene (ring)	0.1813(6)	0.208(1)	0.600(4)	4.61(5)
Butylbenzene (chain)	0.315(2)	0.346(3)	1.282(9)	6.7(3)
Decylbenzene (ring)	0.174(2)	0.212(2)	0.634(4)	4.55(4)
Decylbenzene (chain)	0.581(3)	0.599(3)	1.367(8)	6.5(2)
Decane	0.3686(9)	0.375(3)	0.889(8)	4.7(4)

**Table A3:** (Mean) relaxation rate constants of the longitudinal ($R_{1}$) and transverse ($R_{2}$) relaxation of the deuterated maltenes in absence and presence of the asphaltene.

Deuterated Maltene	Without Asphaltene		With 150 g/L Asphaltene	
	$R_{1}$ [$s^{-1}$]	$R_{2}$ [$s^{-1}$]	${\bar{R}}_{1}$ [$s^{-1}$]	${\bar{R}}_{2}$ [$s^{-1}$]
Decalin-d${}_{18}$	1.827(5)	1.82(2)	4.27(4)	28.0(6)
Cyclohexane-d${}_{12}$	0.617(2)	0.647(4)	1.011(7)	9.1(1)
Naphthalene-d${}_{8}$	1.953(8)	1.94(2)	5.42(7)	11.8(8)
Benzene-d${}_{6}$	0.735(2)	0.733(2)	1.534(5)	18.8(2)
Toluene-d${}_{8}$ (ring)	1.162(3)	1.167(4)	2.56(3)	20.4(3)
Toluene-d${}_{8}$ (CH_3_)	0.2162(7)	0.2192(8)	0.348(2)	4.83(4)
Ethylbenzene-d${}_{10}$ (ring)	1.523(4)	0.854(4)	3.00(3)	17.9(4)
Ethylbenzene-d${}_{10}$ (chain)	0.854(4)	0.847(5)	1.168(5)	5.6(2)
Nonane-d${}_{20}$	1.928(7)	1.89(2)	2.56(2)	7.06(9)

## Appendix D. Fast Transverse Relaxation of Cyclohexane and Decalin

A biexponential fit of the transverse relaxation decay of the decalin protons in the asphaltene-free solution results in one $T_{2}$ component similar to $T_{1}$, and one $T_{2}$ component 20 times shorter than $T_{1}$. In addition, the time constant of the transverse relaxation of the cyclohexane protons is by a factor of ten shorter than $T_{1}$. These observations contradict the expected $T_{1}=T_{2}$ in the extreme narrowing limit. This short $T_{2}$ is a result of an internal motion present in cyclohexane and decalin, known as ring flip [53], which is the interconversion between equivalent ring shapes of cyclic conformers.

This interconversion can be described as chemical exchange between nuclei at two different sites A and B, with the respective resonance frequencies $\omega_{A}$ and $\omega_{B}$ and transverse relaxation times $T_{2A}$ and $T_{2B}$. The exchange takes place at the exchange rate $k_{ex}=k_{A}+k_{B}=\tau_{ex}^{-1}.$ In case of a fast exchange, a single resonance line is observed (e.g., cyclohexane at room temperature), while two lines can be observed for a slow exchange (e.g., cyclohexane at, for instance, −77.9 ${}^{\circ }C$ [54]). In the presence of a chemical exchange, the transverse relaxation time constant obtained by the CPMG pulse sequence depends on the echo time, since the dephasing rate is affected when a nucleus changes its precession frequency from $\omega_{A}$ to $\omega_{B}$ during the exchange from site A to B. If the echo time is much smaller than the exchange time, the dephasing resulting from the exchange is negligible, while in the opposite case it is maximal, and a shortened $T_{2}$ can be observed [55,56]. The transverse relaxation rate constant in the limit of $\tau_{E}\to \infty$ can be computed as follows:

(A1) $$\begin{array}{cc} \; & \lim_{\tau_{E}\to \infty }R_{2}=\frac{\left(T_{2A}+T_{2B}\right)}{2T_{2A}T_{2B}}+\frac{k_{ex}}{2}-\Gamma \end{array}$$

$$\begin{array}{c} \begin{array}{cc} \Gamma =ℜ & [{\left(\frac{\left(T_{2B}-T_{2A}\right)}{2T_{2A}T_{2B}}+\frac{k_{ex}}{2}\left(p_{B}-p_{A}\right)\right)}^{2}-\frac{\Delta \omega^{2}}{4}+k_{ex}^{2}p_{A}p_{B}\\ \; & +i\Delta \omega \left(\frac{\left(T_{2B}-T_{2A}\right)}{2T_{2A}T_{2B}}+\frac{k_{ex}}{2}\left(p_{B}-p_{A}\right)\right){]}^{1/2} \end{array} \end{array}$$

with $p_{A}+p_{B}=1$, where $p_{A}$ and $p_{B}$ denote the equilibrium population of spins in sites A and B. Ring flips occur in the chair conformation of cyclohexane and the *cis* conformation of decalin, but not its *trans* conformation due to steric constraints. Since $\tau_{e}=1\;\mathrm{ms}\;\gg \;\tau_{ex}(C6H12)\approx 14\;\mu s$ (see Table A4), the shortening of $T_{2}$ can be expected. Furthermore, the chair conformation of cyclohexane is slightly more stable than the twist-boat conformation, so that the first is the dominant conformation at room temperature [53] and only one time constant for the relaxation is observed. The biexponential decay of decalin then simply results from the coexistence of a mixture of *cis* and *trans* decalin. Since the dynamic of the ring flip does not differ much for the protonic and the deuterated variants, see, for example, the exchange rate of C_6_H_12_ and C_6_D_12_ in Table A4, the shortened $T_{2}$ is in principle also observable for cyclohexane-d${}_{12}$ and decalin-d${}_{18}$. Due to the more than six times smaller gyromagnetic ratio of ${}^{2}$H, the chemical shift difference diminishes, and the shortening of $T_{2}$ becomes negligible at the used magnetic field strength, as one can see in Table A4 by comparing the observed $T_{1}$ with the short limit of $T_{2}$.

**Table A4:** Estimation of the short limit of $T_{2}$ of cyclohexane-h${}_{12}$/-d${}_{12}$ in case of $\tau_{E}\to \infty$, according to Equation (A1). Since the asphaltene-free solution should fulfill the extreme narrowing limit, where $T_{1}=T_{2}$, $T_{2A}=T_{2B}\approx T_{1}$ is assumed for the transverse relaxation time constants of the nuclei at the different sites. The exchange rates correspond to a sample temperature of ∼ 20 ${}^{\circ }C$ and were taken from references [57] (C_6_H_12_) and [58] (C_6_D_12_) (determined for cyclohexane-h${}_{12}$/-d${}_{12}$ in a solution of liquid crystals). The chemical shift differences were taken from references [54] (C_6_D_11_H at −100 ${}^{\circ }C$) and [59] (neat plastic-crystalline C_6_D_12_ at −80 ${}^{\circ }C$).

Parameter	Cyclohexane	Cyclohexane-d${}_{12}$
$T_{1}$	6.96 s	1.621 s
$p_{A}=p_{B}$	0.5	0.5
$k_{ex}$	72,885 s^−1^	105,390 s^−1^
Δω	0.462 ppm	0.5 ppm
	871 Hz	145 Hz
$\lim_{\tau_{E}\to \infty }T_{2}$	0.364 s	1.5 s
